# Seroprevalence of hepatitis B virus (HBV) and hepatitis C virus (HCV) among human immunodeficiency virus (HIV)-infected patients in an HBV endemic area in Brazil

**DOI:** 10.1371/journal.pone.0203272

**Published:** 2018-09-07

**Authors:** Claudinei Mesquita da Silva, Leyde Daiane de Peder, Alessandra Michele Guelere, Josana Dranka Horvath, Eraldo Schunk Silva, Jorge Juarez Vieira Teixeira, Dennis Armando Bertolini

**Affiliations:** 1 Post-graduate Program in Health Sciences, Maringá State University, Maringá, Paraná, Brazil; 2 Post-graduate Program in Bioscience and Physiopathology, Maringá State University, Maringá, Paraná, Brazil; 3 Post-graduate University Center of Assis Gurgacz Foundation, Cascavel, Paraná, Brazil; 4 Specialized Center of Infectious and Parasitic Diseases, Cascavel, Paraná, Brazil; 5 Department of Statistics, Maringá State University, Maringá, Paraná, Brazil; 6 Department of Clinical Analysis and Biomedicine, Maringá State University, Maringá, Paraná, Brazil; University of Cincinnati College of Medicine, UNITED STATES

## Abstract

**Background:**

Hepatitis B virus (HBV) and hepatitis C virus (HCV) are a common cause of complications in liver disease and immunological impairment among human immunodeficiency virus (HIV)-infected patients. The aim of this study was to assess the seroprevalence of HBV and HCV and their correlation with CD4+ T-cells among HIV-infected patients in an HBV endemic area.

**Methods:**

A cross-sectional observational and retrospective study was carried out in a reference center in Southern Brazil between January 2005 and December 2016. Socio-demographic data were collected by using a structured questionnaire. Serological tests and analysis of CD4+ T-cell count levels were performed using standard procedures.

**Results:**

The seroprevalence of HIV-HBV, HIV-HCV, and HIV-HBV-HCV coinfections was 3.10%, 3.10%, and 0.16%, respectively. At baseline, anti-hepatitis B surface and anti-hepatitis B core antigens were detected in 46.27% and 16.74% of HIV-monoinfected patients and in 31.25% and 21.86% of the HIV-HCV coinfected patients, respectively. The median CD4+ T-cell count at baseline in the HIV-monoinfected group was higher than that in the HIV-coinfected groups, but without statistical significance. The median CD4+ T-cell count and the CD4/CD8 ratio were significantly higher in HIV-HBV and HIV-HCV groups after 24 months of combination antiretroviral therapy (cART) compared to the pre-cART values. When comparing patients with HIV-HBV and HIV-HCV on cART, CD4+ T-cell recovery was more rapid for HIV-HBV patients.

**Conclusion:**

Although the analyzed region was endemic for HBV, the prevalence of HIV-HBV and HIV-HCV coinfection was lower than the rate found in the general population of Brazil. HBV and HCV had no significant impact on CD4+ T-cell counts among HIV-infected patients at baseline.

## Introduction

Human immunodeficiency virus (HIV), hepatitis B virus (HBV), and hepatitis C virus (HCV) are among the leading causes of death by infectious diseases worldwide [1]. Globally, in 2015, approximately 36.7 million people were living with HIV infection [2], 257 million people with chronic HBV infection, and 71 million people with HCV infection [3]. These viruses have similar routes of transmission, and it is estimated that 2.7 and 2.3 million people are living with HIV-HBV and HIV-HCV coinfection, respectively [3].

Comorbidities such as chronic liver disease caused by HBV or HCV infection are recognized as significant problems in HIV-infected patients [4]. Evidence suggests that coinfection with HBV or HCV adversely affects the clinical course of HIV infection [5,6]. Furthermore, HIV-HBV coinfections, especially those with a high HBV DNA load, are associated with lower CD4+ T-cell counts before treatment initiation [7]. Similarly, HIV-HCV coinfection has emerged as an important cofactor that should be considered in immunological progression of HIV infection and immunological response [8]. However, in other studies, HBV or HCV have not demonstrated an impact on the disease progression among HIV-infected patients [9–11].

Immunosuppression caused by HIV infection has been associated with the increased progression of hepatic diseases as well as increased risk of chronic infection with HBV or HCV [4]. In addition, a body of evidence suggests that in HIV infection, HBV and HCV infections, among other opportunistic infections, are associated with CD4+ T-cell counts reducing to values below normal [5,7,12,13]. Additionally, combination antiretroviral therapy (cART) and HBV or HCV reactivation are associated with hepatotoxicity in HIV-coinfected patients, rendering them more susceptible to liver-related diseases and impaired CD4+ T-cell recovery [14,15]. However, patients with liver disease, through splenic sequestration of lymphocyte, may lead to a discrepancy between absolute CD4+ T-cell counts and CD4+ T-cell percentage, obscuring the accurate interpretation of these values [16].

Studies reported that the rates of coinfection of HIV with either HCV or HBV vary from region to region and based on study population and risk factors for virus acquisition [17,18]. In Brazil, studies showed that among HIV-infected individuals, approximately 3.8–28.8% were positive for HBV; as assessed by the hepatitis B surface antigen (HBsAg) test, while HCV positivity (detected by the anti-HCV antibody test) varied from 9.7 to 53.8% [19–21]. However, the epidemiological and clinical profiles of HBV and/or HCV infection among HIV-infected patients remain unknown in many regions of Brazil [22]. Further, the impact of HBV or HCV on immunologic parameters of HIV-infected persons is poorly characterized. Thus, the aim of this study was to assess the seroprevalence of HBV and HCV in Southern Brazil, which is endemic for HBV [23], its correlation with CD4+ T-cells among HIV-infected patients at baseline, and the effect of cART on CD4+ T-cell count recovery in HIV-coinfected patients.

## Materials and methods

### Study area

This study was conducted at the Specialized Center for Infectious and Parasitic Diseases, CEDIP (Centro Especializado de Doenças Infecto Parasitárias), Cascavel city, Paraná State, Brazil. CEDIP serves 25 municipalities within the 10^th^ Health Region of the State of Paraná, which comprises a population of 502,591 individuals [24] and is part of the Unified Health System (Sistema Único de Saúde). The treatment program for patients with HIV infection at this reference center consists of follow-up visits with health care professionals for treatment pickup and monitoring of the HIV viral load level, CD4+ and CD8+ T-cell counts; other laboratory indices (e.g., liver and kidney function and lipids) and clinical data are also recorded.

### Study design

We developed a cross-sectional observational and retrospective study with data collection between January 1, 2005 and December 31, 2016. Socio-demographic information and other relevant possible risk factors of the enrolled participants were collected by trained nurses and physicians, using a structured and pre-tested questionnaire. The following variables were considered: date of birth, sex, marital status, ethnicity, level of educational attainment, sexual preference (heterosexual or homosexual/bisexual), date of HIV infection diagnosis, form of HIV infection, alcohol consumption, use of injection drugs, and presence of tattoos. Baseline laboratory testing (first measurements after a positive anti-HIV test) included HBsAg, anti-HCV, HCV RNA, anti-HBs, anti-hepatitis B core (HBc), CD4+ T-cell count, and HIV viral load were also included. Data from the first 24 months of regular visits of HIV-HBV and HIV-HCV coinfected patients receiving cART were collected, including HIV viral load, CD4+ T-cell count, and CD4/CD8 ratio.

### Participants

All HIV-infected patients, and who were tested at baseline for HBV and HCV were included in the study. To be enrolled in this follow-up, HIV-infected patients had to meet the following requirements: (1) age ≥ 18 years old, (2) positive test for HBV or HCV at baseline, (3) have received cART for at least 24 months, and (4) be cART-naïve at baseline. For those who met the inclusion criteria, measurements of CD4+ T-cell count, CD4/CD8 ratio, and HIV viral load were collected retrospectively collected to obtain baseline (prior to cART) then 12 and 24 months of cART.

Participants were defined as presenting HBV infection if they tested positive for HBsAg and negative for anti-HCV or positive for HBsAg and anti-HCV and negative for HCV RNA (HIV-HBV). Participants testing negative for HBsAg and positive for both anti-HCV and HCV RNA were defined as HCV-infected (HIV-HCV). Participants testing positive for HBsAg, anti-HCV, and HCV RNA were grouped as triply infected (HIV-HBV-HCV). Participants were identified as HIV-monoinfected when they tested negative for both HBsAg and HCV RNA. For the purposes of calculation, the study population was divided into the following four groups: HIV-monoinfected, HIV-HBV, HIV-HCV, and all HIV-coinfected (HIV-HBV + HIV-HCV + HIV-HBV-HCV).

This study was reviewed and approved by CEDIP and the Research Ethical Committee of the University Center of Assis Gurgacz Foundation (Report N^o^ 1.397.212 of 28/01/2016). The waiver of the consent of patients by the Ethics Committee was because the research occurred with information from a secondary database. At the same time, the research team did not maintain contact with the interviewees at any time during the study. The norms of ethical research were followed by the researchers according to the requirements of the country, guaranteeing total confidentiality and anonymity of the data.

### Laboratory measurements

HIV infection status was based on the positive test results of an HIV enzyme-linked immunosorbent assay (Abbott Diagnostics, Chicago, USA) from two peripheral blood samples and was confirmed by western blotting (Bio-Rad, Marnes La Coquette, France). HBsAg, anti-HBs, anti-HBc, and anti-HCV were tested by commercially available enzyme immunoassay (Abbott Diagnostics). Positive results for the presence of anti-HCV were confirmed by amplification of HCV RNA using reverse transcription-polymerase chain reaction (RT-PCR) by COBAS Ampliprep/Cobas TaqMan48 real-time RT-PCR (Roche Diagnostics), as described elsewhere [25]. The CD4+ and CD8+ T-cell counts were estimated with flow cytometry (BD Trucount Tubes) using the FACSCalibur apparatus (Becton-Dickinson, New Jersey, USA) and the results were expressed in cells/mm^3^. HIV RNA levels were measured using real-time polymerase chain reaction (Roche Diagnostics, GmbH, Mannheim, Germany) with a detection limit of 50 copies/ml. Data of CD4+ T-cell count, CD4/CD8 ratio, and HIV viral load were stored and subsequently obtained for tabulation of data from the national network of the Sistema de Controle de Exames Laboratoriais (SISCEL), the Laboratory Test Control System, at the virology laboratory of the State University of Maringá. All information from SISCEL is stored using data encryption in its central database, which is located in the Department of Surveillance, Prevention and Control of STIs, HIV/AIDS and Viral Hepatitis of the Ministry of Health and can be accessed online.

### Data analysis

The prevalence of HBV and HCV coinfections among HIV-infected patients was analyzed by descriptive statistics and presented as percentages. Chi-squared and Fisher’s exact tests were used to determine the relationship between categorical variables, while Wilcoxon test was used to analyze the relationship between continuous variables. Multivariate logistic analysis was used to determine risk factors. Odds ratio (OR) with 95% confidence interval and p-value were calculated. The level of significance was set at p<0.05. All data were analyzed using the Statistical Analysis Software (SAS) software (Statistical Analysis Software, Cary, North Carolina, EUA) version 9.4 [26], except that multivariate logistic analysis was conducted using Stata version 12.0 [27].

## Results

Between January 2005 and December 2016, 1,717 patients were diagnosed with HIV infection in the region under study. Among them, 1,029 (59.93%) were men, with a median age of 32 years and interquartile range (IQR) of 24–42; and 688 (40.07%) were women, with a median age of 33 years (IQR 25–43) (p = 0.399). Among the HIV-infected patients, 1,259 (73.33%) were tested for HBV (HBsAg) and HCV (anti-HCV). In this population, 58.06% (731/1,259) were men, with a median age of 31 years (IQR 24–41); and 41.94% (528/1,259) were women, with a median age of 32 years (IQR 25–42) (p = 0.237). The overall prevalence of HIV-coinfected was 80/1,259 (6.36%), with a prevalence of HIV-HBV, HIV-HCV, and HIV-HBV-HCV coinfections of 39 (3.10%), 39 (3.10%), and 2 (0.16%), respectively. Among all HIV-coinfected patients, 51 (63.75%) were male (median age of 35 years; IQR 28–44) and 29 (36.25%) were female (median age of 33 years; IQR 25–39) (p = 0.184), with a male to female ratio of 1.76:1. Patients aged between 30 and 49 years of the male sex, single, from a white ethnic background, heterosexual, with a sexually transmitted form of HIV infection, and having no tattoos were predominant among all the studied groups; however, no statistical association was found. Nevertheless, compared to HIV-monoinfected group, HIV-HCV and all HIV-coinfected groups were more likely to be injecting drug users (IDU), excessive alcohol consumers, and had a period of more than 5 years of positive HIV diagnosis (p< 0.05) (Table 1).

**Table 1:** Baseline characteristics of HIV-monoinfected and HIV-coinfected patients.

Variables	HIV-monoinfected n (%)	HIV-HBV n (%)	^a^p- value	HIV-HCV n (%)	^b^p- value	All HIV-coinfected n (%)	^c^p- value	^d^p-value
**Age**								
0–17	55 (4.68)	0 (0.00)	0.089	2 (5.13)	0.992	2 (2.50)	0.312	0.196
18–29	476 (40.51)	10 (25.64)		16 (41.02)		26 (32.50)		
30–49	505 (42.98)	23 (58.98)		17 (43.59)		42 (52.50)		
≥ 50	139 (11.83)	6 (15.38)		4 (10.26)		10 (12.50)		
**Sex**								
Male	680 (57.68)	22 (56.41)	1.000	27 (69.23)	0.203	51 (63.75)	0.343	0.349
Female	499 (42.32)	17 (43.59)		12 (30.77)		29 (36.25)		
**Marital status**								
Single	562 (47.99)	18 (46.15)	0.571	18 (48.65)	0.855	36 (46.75)	0.556	0.649
Married	459 (39.20)	17 (43.59)		16 (43.24)		34 (44.16)		
Divorced	100 (8.54)	4 (10.26)		2 (5.41)		6 (7.79)		
Widowed	50 (4.27)	0 (0.00)		1 (2.70)		1 (1.30)		
**Ethnicity**								
White	704 (61.11)	20 (55.55)	0.892	24 (64.86)	0.654	45 (60.00)	0.866	0.303
Black	42 (3.65)	2 (5.56)		0 (0.00)		2 (2.67)		
Brown	400 (34.72)	14 (38.89)		13 (35.14)		28 (37.33)		
Other	6 (0.52)	0 (0.00)		0 (0.00)		0 (0.00)		
**Education**								
≤ 8 years	551(48.72)	25 (65.79)	0.057	22 (61.11)	0.195	48 (64.00)	0.015	0.860
> 8 years	580 (51.28)	13 (34.21)		14 (38.89)		27 (36.00)		
**Sexual preference**								
Heterosexual	880 (77.13)	32 (86.49)	0.254	32 (86.49)	0.254	66 (86.84)	0.067	NA
Homosexual/Bisexual	261 (22.87)	5 (13.51)		5 (13.51)		10 (13.16)		
**HIV transmission**								
Sexual	1144 (97.69)	38 (100.00)	0.697	38 (100.00)	0.697	78 (100.00)	0.340	NA
Others	27 (2.31)	0 (0.00)		0 (0.00)		0 (0.00)		
**IDU**								
Yes	8 (0.72)	0 (0.00)	0.609	3 (9.09)	< 0.001	3 (4.23)	0.019	0.208
No	1104 (99.28)	36 (100.00)		30 (90.91)		68 (95.77)		
**Alcohol use**								
Yes	205 (18.55)	9 (23.68)	0.558	13 (40.63)	0.004	22 (30.56)	0.019	0.207
No	900 (81.45)	29 (76.32)		19 (59.38)		50 (69.44)		
**Tattoo**								
Yes	159 (13.89)	3 (8.82)	0.554	3 (9.38)	0.638	6 (8.82)	0.317	0.706
No	986 (86.11)	31 (91.18)		29 (90.63)		62 (91.18)		
**Period HIV diagnosis**								
< 3 years	424 (35.96)	11 (28.20)	0.507	3 (7.69)	< 0.001	14 (17.50)	< 0.001	0.033
3–5 years	227 (19.25)	7 (17.95)		5 (12.82)		12 (15.00)		
> 5 years	528 (44.79)	21 (53.85)		31 (79.49)		54 (67.50)		

HIV, human immunodeficiency virus; HBV, hepatitis B virus; HCV, hepatitis C virus; n, number of patients; NA, not applicable; Fisher’s exact test and Pearson’s Chi-square test for comparison between groups

^a^p-value for HIV-monoinfected *versus* HIV-HBV

^b^p-value for HIV-monoinfected *versus* HIV-HCV

^c^p-value for HIV-monoinfected *versus* all HIV-coinfected

^d^p-value for HIV-HBV *versus* HIV-HCV.

Risk factors for HBV coinfection were analyzed by multivariate logistic analysis. Age was not associated with HBV infection in HIV-infected patients. However, education ≤ 8 years was an independent risk factor for HBV coinfection (OR 2.02; CI 1.02–4.00; p = 0.039). Risk factors for HCV infection among HIV-infected patients were also analyzed. IDU (OR 13.80; CI 3.44–53.44; p<0.001) and period since HIV diagnosis > 5 years (OR 8.29; CI 2.49–27.63; p<0.001) were independent risk factors for HCV coinfection (Table 2).

**Table 2:** Multivariate logistic analysis of HIV-HBV and HIV-HCV patients.

	OR	95% CI	p-value
**Parameters HIV-HBV**			
**Age**			
≥ 50	1	-	**-**
18–29	2.05	0.73–5.77	0.162
30–49	0.95	0.38–2.37	0.909
**Education**			
> 8 years	1	-	-
≤ 8 years	2.02	1.02–4.00	0.039
**Parameters HIV-HCV**			
**Sex**			
Female	1	-	-
Male	1.65	0.83–3.29	0.150
**IDU**			
No	1	-	-
Yes	13.80	3.44–53–44	< 0.001
**Education**			
> 8 years	1	-	-
≤ 8 years	1.65	0.84–3.27	0.143
**Period since HIV diagnosis**			
< 3 years	1	-	-
3–5 years	3.11	0.73–13.20	0.104
> 5 years	8.29	2.49–27.63	< 0.001

HIV, human immunodeficiency virus; HBV, hepatitis B virus; HCV, hepatitis C virus; IDU, injecting drug user; OR, odds ratio; CI, confidence interval.

Among all HIV-infected patients subjected to laboratory tests to determine HBV and HCV infection at baseline, 93.57% (1,103 HIV-monoinfected, 37 HIV-HBV, 36 HIV-HCV, and 2 HIV-HBV-HCV) were subjected to CD4+ T-cell counts. In the HIV-monoinfected group, the median CD4+ T-cell count in females was higher than in males (451 cells/mm^3^; IQR 266–689 cells/mm^3^ *versus* 379 cells/mm^3^; IQR 213–538 cells/mm^3^, respectively; p<0.001). The same was true for the HIV-HBV group (475 cells/mm^3^; IQR 384–591 cells/mm^3^ *versus* 336 cells/mm^3^; IQR 258–477 cells/mm^3^, respectively; p = 0.066), and HIV-HCV group (410 cells/mm^3^; IQR 185–506 cells/mm^3^ *versus* 345 cells/mm^3^; 243–522 cells/mm^3^, respectively; p = 0.525). The highest and the lowest median CD4+ T-cell counts in all patients were observed in the age groups of ≤18 years (582 cells/mm^3^; IQR 423–764 cells/mm^3^) and ≥50 years (333 cells/mm^3^; IQR 168–507 cells/mm^3^), respectively; p<0.001. The median CD4+ T-cell count in the HIV-monoinfected group was higher than that in the HIV-HBV group, but without statistical significance (p = 0.925). A similar situation occurred when the HIV-monoinfected group was compared to the HIV-HCV group (p = 0.460) and with the all HIV-coinfected groups (p = 0.671). The results are summarized in Table 3.

**Table 3:** CD4+ T-cell count and HIV viral load and their association with HBV and/or HCV in HIV-infected patients at baseline.

Groups	CD4+ T-cell count (cells/mm^3^) median (IQR)	p-value	HIV viral load (log copies/ml) median (IQR)	p- value	CD4+ T-cell count (cells/mm^3^)			
					< 200 n (%)	200–500 n (%)	> 500 n (%)	p- value
**HIV-monoinfected**	405 (234–608)		4.10 (3.64–4.45)		232 (21.03)	473 (42.88)	398 (36.08)	
**HIV-HBV**	387 (287–557)	0.925^a^	4.12 (3.17–4.50)	0.675^a^	5 (13.51)	20 (54.06)	12 (32.43)	0.337^a^
**HIV-HCV**	378 (229–514)	0.460^b^ 0.357^c^	4.24 (3.77–4.81)	0.120^b^ 0.137^c^	6 (16.67)	20 (55.55)	10 (27.78)	0.314^b^ 0.879^c^
**All HIV-coinfected**	387 (257–522)	0.671^d^	4.12 (3.47–4.70)	0.559^d^	11 (14.67)	42 (56.00)	22 (29.33)	0.077^d^

HIV, human immunodeficiency virus; HBV, hepatitis B virus; HCV, hepatitis C virus; n, number of patients; IQR, interquartile range

^a^p-value for HIV-monoinfected *versus* HIV-HBV

^b^p-value for HIV-monoinfected *versus* HIV-HCV

^c^p-value for HIV-HBV *versus* HIV-HCV.

^d^p-value for HIV-monoinfected *versus* all HIV-coinfected

The serological markers anti-HBs and anti-HBc were detected in 46.27% (521/1,126) and 16.74% (181/1,081) of HIV-monoinfected patients and in 31.25% (10/32) and 21.86% (7/32) of the HIV-HCV coinfected patients, respectively. Among all the HIV-monoinfected and HIV-HCV coinfected patients who tested positive for anti-HBs, 12.53% (132/1,054) and 30% (3/10), respectively, also tested positive for anti-HBc.

Among the all HIV-coinfected patients, 58.75% (24 HIV-HBV and 23 HIV-HCV) satisfied the inclusion criteria for the retrospective study. After 12 months on cART, there was an increase in CD4+ T-cells in all groups. However, this increase was not significant in the HIV-HCV group. At the end of 24 months of cART, there was a significant increase in the CD4+ T-cell counts in all studied groups. Among those patients who had started cART, 58.3% of the HBV-infected and 83.3% of the HCV-infected patients achieved full HIV viral load suppression (defined as HIV RNA viral load below 50 copies/ml) after 12 months of cART. After 24 months on cART, 66.6% of the patients coinfected with HBV achieved complete suppression of HIV viral load. However, those coinfected with HCV remained at the same level (83.3%). After 12 months of cART, a significant difference in HIV viral load suppression was observed in all study groups (Table 4).

**Table 4:** Effect of cART on CD4+ T-cell count, CD4/CD8 ratio and HIV viral load in HIV-HBV and HIV-HCV coinfection.

Groups	cART regimen	CD4+ T-cell count (cells/mm^3^) median (IQR)	p-value	CD4/CD8 ratio median (IQR)	p-value	Log HIV Viral load (copies/ml) median (IQR)	p-value	CD4+ T-cell count (cells/mm^3^)			
								< 200 n (%)	200–500 n (%)	> 500 n (%)	p-value
**HIV-HBV**	Pre-cART	316 (227–466)	0.005^a^	0.30 (0.19–0.59)	0.024^a^	4.25 (2.65–5.10)	< 0.001^a^	5 (20.84)	15 (62.50)	4 (16.66)	0.026^a^
**HIV-HCV**		304 (154–478)	0.085^a^	0.32 (0.16–0.47)	0.035^a^	4.05 (1.70–5.35)	< 0.001^a^	6 (26.09)	13 (56.52)	4 (17.39)	0.118^a^
**HIV-HBV + HIV-HCV**		312 (202–467)	0.004^a^	0.30 (0.17–0.50)	0.006^a^	3.56 (1.70–4.21)	< 0.001^a^	11 (23.41)	28 (59.57)	8 (17.02)	0.006^a^
**HIV-HBV**	On-cART (12 months)	512 (346–613)	0.042^b^	0.48 (0.30–0.70)	0.051^b^	1.70 (1.70–1.90)	0.012^b^	1 (4.17)	11 (45.83)	12 (50.00)	0.482^b^
**HIV-HCV**		344 (252–556)	0.008^b^	0.48 (0.29–0.56)	0.045^b^	1.70 (1.70–1.70)	0.076^b^	1 (4.35)	16 (69.57)	6 (26.08)	0.219^b^
**HIV-HBV + HIV-HCV**		435 (266–569)	0.007^b^	0.48 (0.29–0.63)	0.008^b^	1.70 (1.70–1.78)	0.004^b^	2 (4.26)	27 (57.45)	18 (38.29)	0.172^b^
**HIV-HBV**	On-cART (24 months)	613 (423–748)	< 0.001^c^	0.66 (0.48–0.89)	< 0.001^c^	1.70 (1.40–1.70)	< 0.001^c^	1 (4.17)	7 (29.17)	16 (66.66)	0.020^c^
**HIV-HCV**		485 (406–561)	< 0.001^c^	0.50 (0.43–0.71)	0.002^c^	1.70 (1.40–1.70)	< 0.001^c^^c^	0 (0.00)	12 (52.17)	11 (47.83)	0.010^c^
**HIV-HBV + HIV-HCV**		554 (406–662)	< 0.001^c^	0.61 (0.46–0.86)	< 0.001^c^	1.70 (1.40–1.70)	< 0.001^c^	1 (2.13)	19 (40.43)	27 (57.44)	< 0.001^c^

cART, combination antiretroviral therapy; HIV, human immunodeficiency virus; HBV, hepatitis B virus; HCV, hepatitis C virus; n, number of patients; IQR, interquartile range

^a^p-value for baseline *versus* 12 months on-cART

^b^p-value for 12 *versus* 24 months on-cART

^c^p-value for baseline *versus* 24 months on-cART.

## Discussion

The seroprevalence of HBV and HCV was estimated in an HIV-infected population in Southern Brazil, showing predominance of coinfection in males whose exposure route to HIV was mostly sexual. We observed an overall prevalence of 6.36% for HBV and/or HCV infection among HIV-positive patients during the period studied. The prevalence of HBV and/or HCV among HIV-infected patients and distribution patterns continue to vary across geographical locations with high prevalence being detected among high-risk populations [17,28]. In our study, we found a low prevalence of risk groups. This may explain the low prevalence of coinfection in the present study, despite the high prevalence of HBV in the region studied [23]. In addition, hidden HBV infection was not investigated in this study; therefore, it is possible that the prevalence of HBV-infected patients was underestimated [29]; or the HBV infection was cured, i.e., patients had been infected with HBV and had been cured of it before being exposed to HIV.

In Brazil, the Ministry of Health currently recommends HBV vaccine for the entire population, regardless of age, and recommends it for newborns within the first 12 h of life [30]. The schedule of administration generally involves three doses, with a 30-day interval between the first and second doses and 6 months between the first and third doses. In Brazil, HIV-positive children as well as adolescents and adults infected with HIV should receive the vaccine for HBV, regardless of the vulnerability conditions [30]. In our study, because of the unavailability of HBV vaccine data at baseline and the complexity of the HBV vaccination program, it was not possible to establish whether the vaccination program contributed to the low prevalence of HBV infection among HIV-infected patients. However, we emphasize the need for a systematic vaccination of all patients with or without HIV infection, without anti-HBs and anti-HBc antibodies.

In the current study, the HBV coinfection rate was associated with education ≤ 8 years (OR 2.02; CI 1.02–4.00; p = 0.039). This is probably because patients with a lower level of education tend to access health services such as vaccination and health education less efficiently [31]. Therefore, health education urgently needs to be adjusted to the life-styles of the people living in this region. In addition, injection of drugs was an independent risk factor for HIV-HCV coinfection (OR 13.80; CI 3.44–53.44; p<0.001). HCV transmission occurs predominantly via blood, as explained in a study in China reporting an increased risk of HIV-HCV coinfection in IDU (OR 36.11; CI 23.519–56.122; p<0.001) [28]. In studies performed in Brazil, the use of injectable drugs still exhibited a strong association with HCV positivity among HIV-infected individuals [32,33]. Our study corroborates these results, although the percentage of IDU in the present sample was small. The high rate of HCV infection among the HIV-positive IDUs makes the burden of management of HIV infection an even uphill task, as HCV infection may affect the course and management of HIV infection, as described elsewhere [34].

The prevalence of serological markers of HBV exposure in HIV-infected individuals varies according to the geographical area and the risk rates of the studied population. Although only 3.10% of the HIV-coinfected patients were HBsAg positive at baseline, 16.74% and 21.86% of HIV-monoinfected and HIV-HCV coinfected patients, respectively, were anti-HBc positive, indicating that these HIV-infected patients had also been exposed to HBV. Anti-HBc is frequent in HIV-infected patients [35,36]. Studies in HIV populations reported high rates of anti-HBc in China, Greece, and Cameroon, with 88.1%, 48.1%, and 81.7%, respectively [28,35,37]. Anti-HBc alone in the HIV population can be interpreted as a marker of occult HBV infection [36], a phenomenon that can be caused by a resolved HBV infection [38] or loss of anti-HBs over time [39]. We believe that, in our study, although the prevalence of HBsAg was low in the HIV population, some of HIV-monoinfected and HIV-HCV coinfected patients may have occult HBV infection.

The absolute CD4+ T-cell count is an important prognostic biomarker that can be employed to establish decision points for initiating appropriate therapy in HIV-infected patients [40]. At baseline, patients with HIV-HBV coinfection had lower median CD4+ T-cell count than those of patients with HIV monoinfection. Correspondingly, patients with HCV coinfection had a lower median CD4+ T-cell count compared to HIV-monoinfected patients, both without statistical association. Coinfection with HIV and HBV can cause complex interactions because HIV impairs the cellular immunity, leading to increased replication of HBV, and HBV enhances HIV replication by activation of inflammatory cytokines and transcriptional factor, as described elsewhere [41]. This increase in viral replication of both HIV and HBV can further contribute to the impairment of the immune system [7]. On the other hand, HCV infection can impact the course of HIV infection via chronic immune activation and cytokine production in HIV-coinfected individuals [42,43], which can result in diminished CD4+ T-cell counts [44]. Furthermore, HCV among HIV-infected patients has also been associated with increased CD4+ T-cell apoptosis [45] and can cause damage to the immune system, which can subsequently increase viral replication of HIV and HCV, further contributing to an impaired immune system and consequently lower CD4+ T-cell counts than were those with only HIV [46,47]. However, caution should be used in CD4+ T-cell count interpretation because it may not truly reflect the HIV-infected patient’s immunologic status. Factors as leukopenia, medications, advanced liver disease, splenomegaly, viral infections such as Epstein Barr virus, cytomegalovirus, human T-cell leukemia virus 1, and bacterial infections such as tuberculosis can cause the absolute CD4+ T-cell count to decrease [48].

In our study, there was a significant increase in CD4/CD8 ratio after 12 and 24 months of cART. The CD4/CD8 ratio may provide relevant information in treated and virologically suppressed HIV-infected subjects because it correlates positively with variables that summarize the immunological background CD4+ T-cell nadir and accumulated cART exposure and negatively with activation markers in CD4+ and CD8+ T-cells [49]. We also found that during cART, the declining trend of HIV RNA level was similar among the groups, and there were significant differences between the pre- and post-cART HIV viral loads. The HIV RNA viral loads at baseline were in accordance with those reported in other studies [50,51]. During cART, a significant increase in CD4+ T-cell counts occurred in all studied groups after 24 months on cART. The comparison between HIV-HBV and HIV-HCV patients in relation to the baseline CD4+ T-cell counts and after 12 and 24 months showed a lower CD4+ T-cell recovery in the HIV-HCV coinfected patients. Seminari *et al*. reported that HIV-HCV coinfection impaired early but not late immunological recovery after cART [52]. In addition, increased T-cell apoptosis [53], chronic immune activation, and cytokine T-lymphocyte exhaustion [42,43] can explain impaired immune recovery in HIV-HCV.

The present study has certain limitations. First, the use of HBsAg positivity as the sole indicator of chronic HBV infection can be misleading. To diagnose a chronic HBV infection, it is required that HBsAg remains detectable for longer than 6 months. As only one HBsAg test at baseline was carried out in this study, some cases might have been misclassified. In addition, cases of occult HBV infection (HBV DNA-positive but HBsAg-negative) were ignored. Second, at baseline, tests for CD4+ T-cell counts were not performed on all HIV-infected patients, which restricted our analysis. Third, missing data were another issue we encountered. Fourth, CD4+ T-cell gain was analyzed for HIV-coinfected patients for whom there were baseline and follow-up results and who were on cART, also restricting our analysis. However, we do not believe these limitations affected our estimates.

In conclusion, although the region studied is endemic for HBV, the prevalence of HIV-HBV and HIV-HCV coinfection was lower than the rate found in the general population of Brazil. At baseline, there was no significant difference in CD4+ T-cell count between the HIV-monoinfected and HIV-HBV or HIV-HCV coinfected groups. After 24 months of cART, there was a significant increase in CD4+ T-cell counts and CD4/CD8 ratio among HIV-HBV and HIV-HCV coinfected patients. When comparing patients with HIV-HBV and those with HIV-HCV on cART, CD4+ T-cell recovery was more rapid for HIV-HBV patients. Future studies are needed to fully understand the implications of HBV and HCV coinfection on immune recovery among HIV-infected patients on cART.

## Supporting information

S1 Table Data from HIV-infected patients on baseline. N = 1,717 HIV-infected patients.
(XLSX)

S2 Table Baseline and cART for 12 and 24 months data. N **=** 24 HIV-HBV and 23 HIV-HCV.
(XLSX)

S1 File Statistical report SAS. (PDF)

S2 File Statistical report STATA. Subtitles Stata. Age: ≥ 50 = 0, 18–29 = 2, 30–49 = 1; Education: > 8 years = 0, ≤ 8 years = 1; Sex: Female = 0, Male = 1; IDU: no = 0, yes = 1; Period since HIV diagnosis: <3 years = 2; 3–5 years = 1; > 5 years = 0.
(LOG)
