# Supplementary material for: Seroprevalence of hepatitis B virus (HBV) and hepatitis C virus (HCV) among human immunodeficiency virus (HIV)-infected patients in an HBV endemic area in Brazil
Source: PLoS One. 2018 Sep 7;13(9):e0203272. doi: 10.1371/journal.pone.0203272 (PMC6128547; doi:10.1371/journal.pone.0203272)
Supplement: S1 File — (PDF) [file pone.0203272.s003.pdf]

Statistical Report

All patients diagnosed with HIV during the study period (n=1717)

The FREQ Procedure

sex				
**sex**	**Frequency**	**Percent**	**Cumulative Frequency**	**Cumulative Percent**
**female**	688	40.07	688	40.07
**male**	1029	59.93	1717	100.00

Frequency Missing = 57

Variable: age (age)
sex=male

Moments			
**N**	1018	**Sum Weights**	1018
**Mean**	33.7043222	**Sum Observations**	34311
**Std Deviation**	12.3134257	**Variance**	151.620453
**Skewness**	0.44102847	**Kurtosis**	0.14824806

Quantiles (Definition 5)	
**Quantile**	**Estimate**
**100% Max**	73
**99%**	65
**95%**	57
**90%**	50
**75% Q3**	42
**50% Median**	32
**25% Q1**	24
**10%**	20
**5%**	18
**1%**	0
**0% Min**	0

The UNIVARIATE Procedure
Variable: age (age)
sex=female

Moments			
**N**	678	**Sum Weights**	678
**Mean**	33.939528	**Sum Observations**	23011
**Std Deviation**	13.3768534	**Variance**	178.940208
**Skewness**	0.10574218	**Kurtosis**	0.17126393

Quantiles (Definition 5)	
**Quantile**	**Estimate**
**100% Max**	81
**99%**	65
**95%**	57
**90%**	53
**75% Q3**	43
**50% Median**	33
**25% Q1**	25
**10%**	19
**5%**	16
**1%**	0
**0% Min**	0

The UNIVARIATE Procedure

The NPAR1WAY Procedure

Wilcoxon Scores (Rank Sums) for Variable age Classified by Variable sex

sex	N	Sum of Scores	Expected Under H0	Std Dev Under H0	Mean Score
**female**	678	583619.50	575283.0	9876.14920	860.795723
**male**	1018	855436.50	863773.0	9876.14920	840.310904

Average scores were used for ties.

Wilcoxon Two-Sample Test	
**Statistic**	583619.5000
**Normal Approximation**	
**Z**	0.8441

Wilcoxon Two-Sample Test	
**One-Sided Pr > Z**	0.1993
**Two-Sided Pr > |Z|**	0.3986

All patients who were tested for HBV and HCV (n=1259)

The SAS System

The FREQ Procedure

HIV_HBV	Frequency	Percent	Cumulative Frequency	Cumulative Percent
**non_reactive**	1218	96.90	1218	96.90
**reagent**	39	3.10	1257	100.00

Frequency Missing = 2

HIV_HCV	Frequency	Percent	Cumulative Frequency	Cumulative Percent
**non_reactive**	1218	96.90	1218	96.90
**reagent**	39	3.10	1257	100.00

Frequency Missing = 2

HIV_HBV_HCV	Frequency	Percent	Cumulative Frequency	Cumulative Percent
**non_reactive**	1218	99.84	1218	99.84
**reagent**	2	0.16	1220	100.00

Frequency Missing = 2

hepatitis	Frequency	Percent	Cumulative Frequency	Cumulative Percent
**non_reactive**	1179	93.65	1179	93.65
**reagent**	80	6.35	1259	100.00

The SAS System

The FREQ Procedure
HIV-monoinfected_and_HIV_hepatitis

sex				
**sex**	**Frequency**	**Percent**	**Cumulative Frequency**	**Cumulative Percent**
**female**	528	41.94	528	41.94
**male**	731	58.06	1259	100.00

The SAS System

The UNIVARIATE Procedure
Variable: age (age)
sex=male

Moments			
**N**	731	**Sum Weights**	731
**Mean**	33.1340629		
**Std Deviation**	12.1459651	**Variance**	147.524468
**Skewness**	0.47682548	**Kurtosis**	0.08956363

Quantiles (Definition 5)	
**Quantile**	**Estimate**
**100% Max**	70
**99%**	63
**95%**	56
**90%**	50
**75% Q3**	41
**50% Median**	31
**25% Q1**	24
**10%**	20
**5%**	18
**1%**	0
**0% Min**	0

The SAS System

The UNIVARIATE Procedure
Variable: age (age)
sex=female

Moments			
**N**	524	**Sum Weights**	524
**Mean**	33.7232824		
**Std Deviation**	13.1700799	**Variance**	173.451006
**Skewness**	0.20688551	**Kurtosis**	0.25039397

Quantiles (Definition 5)	
**Quantile**	**Estimate**
**100% Max**	81
**99%**	65
**95%**	56
**90%**	53
**75% Q3**	42
**50% Median**	32
**25% Q1**	25
**10%**	19
**5%**	17
**1%**	0
**0% Min**	0

The SAS System

The NPAR1WAY Procedure

Wilcoxon Scores (Rank Sums) for Variable age Classified by Variable sex

sex	N	Sum of Scores	Expected Under H0	Std Dev Under H0	Mean Score
**female**	524	336564.0	329072.0	6329.47918	642.297710
**male**	731	451576.0	459068.0	6329.47918	617.751026

Average scores were used for ties.

Wilcoxon Two-Sample Test	
**Statistic**	336564.0000
**Z**	1.1836
**One-Sided Pr > Z**	0.1183
**Two-Sided Pr > |Z|**	0.2366

The UNIVARIATE Procedure
Variable: age (age)
hepatitis=reagent sex=male

Moments			
**N**	51	**Sum Weights**	51
**Mean**	35.8823529	**Sum Observations**	1830
**Std Deviation**	10.8658125	**Variance**	118.065882
**Skewness**	0.1261037	**Kurtosis**	-0.6400289

Quantiles (Definition 5)	
**Quantile**	**Estimate**
**100% Max**	58
**99%**	58
**95%**	54
**90%**	52
**75% Q3**	44
**50% Median**	35
**25% Q1**	28
**10%**	23
**5%**	18
**1%**	14
**0% Min**	14

The UNIVARIATE Procedure
Variable: age (age)
hepatitis=reagent sex=female

Moments			
**N**	29	**Sum Weights**	29
**Mean**	32.7931034	**Sum Observations**	951
**Std Deviation**	10.523373	**Variance**	110.741379
**Skewness**	0.68765047	**Kurtosis**	0.08078095

Quantiles (Definition 5)	
**Quantile**	**Estimate**
**100% Max**	60
**99%**	60
**95%**	51
**90%**	47
**75% Q3**	39
**50% Median**	33
**25% Q1**	25
**10%**	20
**5%**	18
**1%**	18
**0% Min**	18

The SAS System

The NPAR1WAY Procedure
hepatitis=reagent

Wilcoxon Scores (Rank Sums) for Variable age Classified by Variable sex

sex	N	Sum of Scores	Expected Under H0	Std Dev Under H0	Mean Score
**female**	29	1041.50	1174.50	99.834206	35.913793
**male**	51	2198.50	2065.50	99.834206	43.107843

Average scores were used for ties.

Wilcoxon Two-Sample Test	
**Statistic**	1041.5000
**Normal Approximation**	
**Z**	-1.3272

Wilcoxon Two-Sample Test	
**One-Sided Pr < Z**	0.0922
**Two-Sided Pr > |Z|**	0.1844

Table 1. Socio-demographic characteristics of HIV-infected patients

The SAS System

The FREQ Procedure

**Frequency**
**Col Pct**

Table of Age of HIV_monoinfected_vs_HIV_HBV			
	**HIV_HBV**	**HIV_monoinfected**	**Total**
**18-29**	10	476	486
	25.64	40.51	
**30-49**	23	505	528
	58.98	42.98	
**0-17**	0	55	55
	0.00	4.68	
**>_50**	6	139	145
	15.38	11.83	
**Total**	39	1175	1214
**Frequency Missing = 45**			

Statistics for Table of transformed_age_1 by HIV_monoinfected_vs_HIV_HBV

Statistic	DF	Value	Prob
**Chi-Square**	3	6.5303	0.0885
**Likelihood Ratio Chi-Square**	3	8.3443	0.0394
**Mantel-Haenszel Chi-Square**	1	1.2403	0.2654
**Phi Coefficient**		0.0733	
**Contingency Coefficient**		0.0731	
**Cramer's V**		0.0733	
**WARNING: 25% of the cells have expected counts less than 5. Chi-Square may not be a valid test.**			

Effective Sample Size = 1214
Frequency Missing = 45

**Frequency**
**Col Pct**

Table of sex by HIV_monoinfected_vs_HIV_HBV			
	**HIV_HBV**	**HIV_monoinfected**	**Total**
**female**	17<br>43.59	499<br>42.32	516
**male**	22<br>56.41	680<br>57.68	702
**Total**	39	1179	1218
**Frequency Missing = 41**			

Statistics for Table of sex by HIV_monoinfected_vs_HIV_HBV

Statistic	DF	Value	Prob
**Chi-Square**	1	0.0248	0.8749
**Likelihood Ratio Chi-Square**	1	0.0247	0.8751
**Continuity Adj. Chi-Square**	1	0.0000	1.0000
**Mantel-Haenszel Chi-Square**	1	0.0247	0.8750
**Phi Coefficient**		0.0045	
**Contingency Coefficient**		0.0045	
**Cramer's V**		0.0045	

Fisher's Exact Test	
**Cell (1,1) Frequency (F)**	17
**Left-sided Pr <= F**	0.6288
**Right-sided Pr >= F**	0.4997
**Table Probability (P)**	0.1285
**Two-sided Pr <= P**	0.8708

Effective Sample Size = 1218
Frequency Missing = 41

Frequency
Col Pct

Table of marital_status by HIV_monoinfected_vs_HIV_HBV			
	HIV_HBV	HIV_monoinfected	Total
divorced	4<br>10.26	100<br>8.54	104
married	17<br>43.59	459<br>39.20	476
single	18<br>46.15	562<br>47.99	580
widower	0<br>0.00	50<br>4.27	50
Total	39	1171	1210
Frequency Missing = 49			

Statistics for Table of marital_status by HIV_monoinfected_vs_HIV_HBV

Statistic	DF	Value	Prob
Chi-Square	3	2.0064	0.5711
Likelihood Ratio Chi-Square	3	3.6042	0.3075
Mantel-Haenszel Chi-Square	1	1.0948	0.2954
Phi Coefficient		0.0407	
Contingency Coefficient		0.0407	
Cramer's V		0.0407	
**WARNING: 25% of the cells have expected counts less than 5. Chi-Square may not be a valid test.**			

Effective Sample Size = 1210
Frequency Missing = 49

Frequency
Col Pct

Table of ethnicity by HIV_monoinfected_vs_HIV_HBV			
	HIV_HBV	HIV_monoinfected	Total
black	2<br>5.56	42<br>3.65	44
brown	14<br>38.89	400<br>34.72	414
other	0<br>0.00	6<br>0.52	6
white	20<br>55.55	704<br>61.11	724
Total	36	1152	1188
Frequency Missing = 71			

Statistics for Table of ethnicity by HIV_monoinfected_vs_HIV_HBV

Statistic	DF	Value	Prob
Chi-Square	3	0.8820	0.8298
Likelihood Ratio Chi-Square	3	1.0181	0.7969
Mantel-Haenszel Chi-Square	1	0.5971	0.4397
Phi Coefficient		0.0272	
Contingency Coefficient		0.0272	
Cramer's V		0.0272	
WARNING: 25% of the cells have expected counts less than 5. Chi-Square may not be a valid test.			

Effective Sample Size = 1188
Frequency Missing = 71

Frequency
Col Pct

Table of transformed_degree_level by HIV_monoinfected_vs_HIV_HBV			
	HIV_HBV	HIV_monoinfected	Total
≤ 8 years	25<br>65.79	551<br>48.72	576
> 8 years	13<br>34.21	580<br>51.28	593
Total	38	1131	1169
Frequency Missing = 90			

Statistics for Table of degree_level by HIV_monoinfected_vs_HIV_HBV

Statistic	DF	Value	Prob
Chi-Square	1	4.2868	0.0384
Likelihood Ratio Chi-Square	1	4.3516	0.0370
Continuity Adj. Chi-Square	1	3.6310	0.0567
Mantel-Haenszel Chi-Square	1	4.2831	0.0385
Phi Coefficient		0.0606	
Contingency Coefficient		0.0604	
Cramer's V		0.0606	

Fisher's Exact Test	
Cell (1,1) Frequency (F)	25
Left-sided Pr <= F	0.9878
Right-sided Pr >= F	0.0278
Table Probability (P)	0.0156
Two-sided Pr <= P	0.0471

Effective Sample Size = 1169
Frequency Missing = 90

Frequency<br>Col Pct

Table of sexual_behavior by HIV_monoinfected_vs_HIV_HBV			
	HIV_HBV	HIV_monoinfected	Total
heterosexual	32<br>86.49	880<br>77.13	912
homosexual/bisexual	5<br>13.51	261<br>22.87	266
Total	37	1141	1178
Frequency Missing = 81			

Statistic	DF	Value	Prob
Chi-Square	1	1.7965	0.1801
Likelihood Ratio Chi-Square	1	2.0103	0.1562
Continuity Adj. Chi-Square	1	1.3009	0.2541
Mantel-Haenszel Chi-Square	1	1.7949	0.1803
Phi Coefficient		0.0391	
Contingency Coefficient		0.0390	
Cramer's V		0.0391	

Fisher's Exact Test	
Cell (1,1) Frequency (F)	32
Left-sided Pr <= F	0.9457
Right-sided Pr >= F	0.1241
Table Probability (P)	0.0698
Two-sided Pr <= P	0.2312

Effective Sample Size = 1178
Frequency Missing = 81

**Frequency**
**Col Pct**

WARNING: 20% of the data are missing.

**Table of way_of_acquiring_virus by HIV_monoinfected_vs_HIV_HBV**			
	**HIV_HBV**	**HIV_monoinfected**	**Total**
**OUTROS**	0	27	27
	0.00	2.31	
**sexual**	38	1144	1182
	100.00	97.69	
**Total**	38	1171	1209
**Frequency Missing = 50**			

Statistics for Table of way_of_acquiring_virus by HIV_monoinfected_vs_HIV_HBV

**Statistic**	**DF**	**Value**	**Prob**
**Chi-Square**	1	0.8962	0.3438
**Likelihood Ratio Chi-Square**	1	1.7444	0.1866
**Continuity Adj. Chi-Square**	1	0.1513	0.6973
**Mantel-Haenszel Chi-Square**	1	0.8954	0.3440
**Phi Coefficient**		-0.0272	
**Contingency Coefficient**		0.0272	
**Cramer's V**		-0.0272	
**WARNING: 25% of the cells have expected counts less than 5. Chi-Square may not be a valid test.**			

**Fisher's Exact Test**	
**Cell (1,1) Frequency (F)**	0
**Left-sided Pr <= F**	0.4182
**Right-sided Pr >= F**	1.0000
**Table Probability (P)**	0.4182
**Two-sided Pr <= P**	1.0000

Effective Sample Size = 1209
Frequency Missing = 50

Frequency

Col Pct

Table of diagnostic_period by HIV_monoinfected_vs_HIV_HBV			
	**HIV_HBV**	**HIV_monoinfected**	**Total**
**>_5**	21<br>53.85	528<br>44.79	549
**3 to 5**	7<br>17.95	227<br>19.25	234
**<_3**	11<br>28.20	424<br>35.96	435
**Total**	39	1179	1218

Frequency Missing = 41

Statistics for Table of diagnostic_period by HIV_monoinfected_vs_HIV_HBV

Statistic	DF	Value	Prob
**Chi-Square**	2	1.3574	0.5073
**Likelihood Ratio Chi-Square**	2	1.3671	0.5048
**Mantel-Haenszel Chi-Square**	1	1.3354	0.2478
**Phi Coefficient**		0.0334	
**Contingency Coefficient**		0.0334	
**Cramer's V**		0.0334	

Effective Sample Size = 1218
Frequency Missing = 41

Frequency

Col Pct

Table of Alcohol by HIV_monoinfected_vs_HIV_HBV			
	**HIV_HBV**	**HIV_monoinfected**	**Total**
**no**	29<br>76.32	900<br>81.45	929
**yes**	9<br>23.68	205<br>18.55	214
**Total**	38	1105	1143
**Frequency Missing = 116**			

Statistics for Table of Alcohol by HIV_monoinfected_vs_HIV_HBV

Statistic	DF	Value	Prob
**Chi-Square**	1	0.6359	0.4252
**Likelihood Ratio Chi-Square**	1	0.5993	0.4389
**Continuity Adj. Chi-Square**	1	0.3433	0.5579
**Mantel-Haenszel Chi-Square**	1	0.6353	0.4254
**Phi Coefficient**		-0.0236	
**Contingency Coefficient**		0.0236	
**Cramer's V**		-0.0236	

Fisher's Exact Test	
**Cell (1,1) Frequency (F)**	29
**Left-sided Pr <= F**	0.2701
**Right-sided Pr >= F**	0.8439
**Table Probability (P)**	0.1140
**Two-sided Pr <= P**	0.4015

Effective Sample Size = 1143
Frequency Missing = 116

Frequency<br>Col Pct	Table of injectable_drugs by HIV_monoinfected_vs_HIV_HBV		
	HIV_HBV	HIV_monoinfected	Total
no	36<br>100.00	1104<br>99.28	1140
yes	0<br>0.00	8<br>0.72	8
Total	36	1112	1148
	Frequency Missing = 111		

Statistics for Table of injectable_drugs by HIV_monoinfected_vs_HIV_HBV

Statistic	DF	Value	Prob
Chi-Square	1	0.2608	0.6096
Likelihood Ratio Chi-Square	1	0.5116	0.4745
Continuity Adj. Chi-Square	1	0.0000	1.0000
Mantel-Haenszel Chi-Square	1	0.2606	0.6097
Phi Coefficient		0.0151	
Contingency Coefficient		0.0151	
Cramer's V		0.0151	
WARNING: 25% of the cells have expected counts less than 5. Chi-Square may not be a valid test.			

Fisher's Exact Test	
Cell (1,1) Frequency (F)	36
Left-sided Pr <= F	1.0000
Right-sided Pr >= F	0.7744
Table Probability (P)	0.7744
Two-sided Pr <= P	1.0000

Effective Sample Size = 1148
Frequency Missing = 111

Frequency<br>Col Pct	Table of tattoo by HIV_monoinfected_vs_HIV_HBV		
	**HIV_HBV**	**HIV_monoinfected**	**Total**
**no**	31<br>91.18	986<br>86.11	1017
**yes**	3<br>8.82	159<br>13.89	162
**Total**	34	1145	1179
	**Frequency Missing = 80**		

Statistics for Table of tattoo by HIV_monoinfected_vs_HIV_HBV

Statistic	DF	Value	Prob
**Chi-Square**	1	0.7141	0.3981
**Likelihood Ratio Chi-Square**	1	0.8000	0.3711
**Continuity Adj. Chi-Square**	1	0.3508	0.5536
**Mantel-Haenszel Chi-Square**	1	0.7135	0.3983
**Phi Coefficient**		0.0246	
**Contingency Coefficient**		0.0246	
**Cramer's V**		0.0246	
**WARNING: 25% of the cells have expected counts less than 5. Chi-Square may not be a valid test.**			

Fisher's Exact Test	
**Cell (1,1) Frequency (F)**	31
**Left-sided Pr <= F**	0.8681
**Right-sided Pr >= F**	0.2907
**Table Probability (P)**	0.1588
**Two-sided Pr <= P**	0.6114

Effective Sample Size = 1179
Frequency Missing = 80

HIV_monoinfected_vs._HIV_HCV

The SAS System

The FREQ Procedure

Frequency<br>Col Pct	Table of transformed_age_1 by HIV_monoinfected_vs_HIV_HCV		
	HIV_HCV	HIV_monoinfected	Total
**18-29**	16<br>41.02	476<br>40.51	492
**30-49**	17<br>43.59	505<br>42.98	522
**0-17**	2<br>5.13	55<br>4.68	57
**>_50**	4<br>10.26	139<br>11.83	143
**Total**	39	1175	1214

Frequency Missing = 45

Statistics for Table of transformed_age_1 by HIV_monoinfected_vs_HIV_HCV

Statistic	DF	Value	Prob
**Chi-Square**	3	0.1012	0.9917
**Likelihood Ratio Chi-Square**	3	0.1042	0.9913
**Mantel-Haenszel Chi-Square**	1	0.0429	0.8359
**Phi Coefficient**		0.0091	
**Contingency Coefficient**		0.0091	
**Cramer's V**		0.0091	

WARNING: 25% of the cells have expected counts less than 5. Chi-Square may not be a valid test.

Effective Sample Size = 1214
Frequency Missing = 45

Frequency<br>Col Pct	Table of sex by HIV_monoinfected_vs_HIV_HCV		
	HIV_HCV	HIV_monoinfected	Total
female	12<br>30.77	499<br>42.32	511
male	27<br>69.23	680<br>57.68	707
Total	39	1179	1218

Frequency Missing = 41

Statistics for Table of sex by HIV_monoinfected_vs_HIV_HCV

Statistic	DF	Value	Prob
Chi-Square	1	2.0697	0.1503
Likelihood Ratio Chi-Square	1	2.1401	0.1435
Continuity Adj. Chi-Square	1	1.6224	0.2028
Mantel-Haenszel Chi-Square	1	2.0680	0.1504
Phi Coefficient		-0.0412	
Contingency Coefficient		0.0412	
Cramer's V		-0.0412	

Fisher's Exact Test	
Cell (1,1) Frequency (F)	12
Left-sided Pr <= F	0.1001
Right-sided Pr >= F	0.9477
Table Probability (P)	0.0478
Two-sided Pr <= P	0.1870

Effective Sample Size = 1218
Frequency Missing = 41

Frequency<br>Col Pct	Table of marital_status by HIV_monoinfected_vs_HIV_HCV		
	HIV_HCV	HIV_monoinfected	Total
divorced	2<br>5.41	100<br>8.54	102
married	16<br>43.24	459<br>39.20	475
single	18<br>48.65	562<br>47.99	580
widower	1<br>2.70	50<br>4.27	51
Total	37	1171	1208

Frequency Missing = 51

Statistics for Table of marital_status by HIV_monoinfected_vs_HIV_HCV

Statistic	DF	Value	Prob
Chi-Square	3	0.7785	0.8546
Likelihood Ratio Chi-Square	3	0.8634	0.8342
Mantel-Haenszel Chi-Square	1	0.0031	0.9559
Phi Coefficient		0.0254	
Contingency Coefficient		0.0254	
Cramer's V		0.0254	

WARNING: 25% of the cells have expected counts less than 5. Chi-Square may not be a valid test.

Effective Sample Size = 1208
Frequency Missing = 51

Frequency<br>Col Pct	Table of ethnicity by HIV_monoinfected_vs_HIV_HCV		
	HIV_HCV	HIV_monoinfected	Total
black	0<br>0.00	42<br>3.65	42
brown	13<br>35.14	400<br>34.72	413
other	0<br>0.00	6<br>0.52	6
white	24<br>64.86	704<br>61.11	728
Total	37	1152	1189

Frequency Missing = 70

Statistics for Table of ethnicity by HIV_monoinfected_vs_HIV_HCV

Statistic	DF	Value	Prob
Chi-Square	3	1.6259	0.6535
Likelihood Ratio Chi-Square	3	3.1176	0.3739
Mantel-Haenszel Chi-Square	1	0.3809	0.5371
Phi Coefficient		0.0370	
Contingency Coefficient		0.0370	
Cramer's V		0.0370	

WARNING: 25% of the cells have expected counts less than 5. Chi-Square may not be a valid test.

Effective Sample Size = 1189
Frequency Missing = 70

Frequency<br>Col Pct	Table of degree_level by HIV_monoinfected_vs_HIV_HCV		
	HIV_HCV	HIV_monoinfected	Total
≤ 8 years	22<br>61.11	551<br>48.72	573
> 8 years	14<br>38.89	580<br>51.28	594
Total	36	1131	1167
	Frequency Missing = 92		

Statistics for Table of degree_level by HIV_monoinfected_vs_HIV_HCV

Statistic	DF	Value	Prob
Chi-Square	1	2.1442	0.1431
Likelihood Ratio Chi-Square	1	2.1585	0.1418
Continuity Adj. Chi-Square	1	1.6770	0.1953
Mantel-Haenszel Chi-Square	1	2.1423	0.1433
Phi Coefficient		0.0429	
Contingency Coefficient		0.0428	
Cramer's V		0.0429	

Fisher's Exact Test	
Cell (1,1) Frequency (F)	22
Left-sided Pr <= F	0.9493
Right-sided Pr >= F	0.0975
Table Probability (P)	0.0467
Two-sided Pr <= P	0.1755

Effective Sample Size = 1167
Frequency Missing = 92

Frequency<br>Col Pct	Table of sexual_behavior by HIV_monoinfected_vs_HIV_HCV		
	HIV_HCV	HIV_monoinfected	Total
heterosexual	32<br>86.49	880<br>77.13	912
homosexual/bisexual	5<br>13.51	261<br>22.87	266
Total	37	1141	1178
Frequency Missing = 81			

Statistics for Table of sexual_behavior by HIV_monoinfected_vs_HIV_HCV

Statistic	DF	Value	Prob
Chi-Square	1	1.7965	0.1801
Likelihood Ratio Chi-Square	1	2.0103	0.1562
Continuity Adj. Chi-Square	1	1.3009	0.2541
Mantel-Haenszel Chi-Square	1	1.7949	0.1803
Phi Coefficient		0.0391	
Contingency Coefficient		0.0390	
Cramer's V		0.0391	

Fisher's Exact Test	
Cell (1,1) Frequency (F)	32
Left-sided Pr <= F	0.9457
Right-sided Pr >= F	0.1241
Table Probability (P)	0.0698
Two-sided Pr <= P	0.2312

Effective Sample Size = 1178
Frequency Missing = 81

WARNING: 21% of the data are missing.

Frequency<br>Col Pct	Table of way_of_acquiring_virus by HIV_monoinfected_vs_HIV_HCV		
	**HIV_HCV**	**HIV_monoinfected**	**Total**
**OUTROS**	0<br>0.00	27<br>2.31	27
**sexual**	38<br>100.00	1144<br>97.69	1182
**Total**	38	1171	1209
	**Frequency Missing = 50**		

Statistics for Table of way_of_acquiring_virus by HIV_monoinfected_vs_HIV_HCV

Statistic	DF	Value	Prob
**Chi-Square**	1	0.8962	0.3438
**Likelihood Ratio Chi-Square**	1	1.7444	0.1866
**Continuity Adj. Chi-Square**	1	0.1513	0.6973
**Mantel-Haenszel Chi-Square**	1	0.8954	0.3440
**Phi Coefficient**		-0.0272	
**Contingency Coefficient**		0.0272	
**Cramer's V**		-0.0272	
**WARNING: 25% of the cells have expected counts less than 5. Chi-Square may not be a valid test.**			

Fisher's Exact Test	
**Cell (1,1) Frequency (F)**	0
**Left-sided Pr <= F**	0.4182
**Right-sided Pr >= F**	1.0000
**Table Probability (P)**	0.4182
**Two-sided Pr <= P**	1.0000

Effective Sample Size = 1209
Frequency Missing = 50

Frequency<br>Col Pct	Table of diagnostic_period by HIV_monoinfected_vs_HIV_HCV		
	HIV_HCV	HIV_monoinfected	Total
> 5 years	31<br>79.49	528<br>44.79	559
3-5 years	5<br>12.82	227<br>19.25	232
>5 years	3<br>7.69	424<br>35.96	427
Total	39	1179	1218

Frequency Missing = 41

Statistics for Table of diagnostic_period by HIV_monoinfected_vs_HIV_HCV

Statistic	DF	Value	Prob
Chi-Square	2	19.3328	<.0001
Likelihood Ratio Chi-Square	2	21.6126	<.0001
Mantel-Haenszel Chi-Square	1	18.7505	<.0001
Phi Coefficient		0.1260	
Contingency Coefficient		0.1250	
Cramer's V		0.1260	

Effective Sample Size = 1218
Frequency Missing = 41

Frequency<br>Col Pct	Table of Alcohol by HIV_monoinfected_vs_HIV_HCV		
	HIV_HCV	HIV_monoinfected	Total
no	19<br>59.38	900<br>81.45	919
yes	13<br>40.63	205<br>18.55	218
Total	32	1105	1137

Frequency Missing = 122

Statistics for Table of Alcohol by HIV_monoinfected_vs_HIV_HCV

Statistic	DF	Value	Prob
Chi-Square	1	9.7774	0.0018
Likelihood Ratio Chi-Square	1	8.0795	0.0045
Continuity Adj. Chi-Square	1	8.4049	0.0037
Mantel-Haenszel Chi-Square	1	9.7688	0.0018
Phi Coefficient		-0.0927	
Contingency Coefficient		0.0923	
Cramer's V		-0.0927	

Fisher's Exact Test	
Cell (1,1) Frequency (F)	19
Left-sided Pr <= F	0.0037
Right-sided Pr >= F	0.9989
Table Probability (P)	0.0026
Two-sided Pr <= P	0.0046

Effective Sample Size = 1137
Frequency Missing = 122

Frequency<br>Col Pct	Table of injectable_drugs by HIV_monoinfected_vs_HIV_HCV		
	HIV_HCV	HIV_monoinfected	Total
no	30<br>90.91	1104<br>99.28	1134
yes	3<br>9.09	8<br>0.72	11
Total	33	1112	1145
	Frequency Missing = 114		

Statistics for Table of injectable_drugs by HIV_monoinfected_vs_HIV_HCV

Statistic	DF	Value	Prob
Chi-Square	1	23.6061	<.0001
Likelihood Ratio Chi-Square	1	9.0899	0.0026
Continuity Adj. Chi-Square	1	15.6274	<.0001
Mantel-Haenszel Chi-Square	1	23.5854	<.0001
Phi Coefficient		-0.1436	
Contingency Coefficient		0.1421	
Cramer's V		-0.1436	
WARNING: 25% of the cells have expected counts less than 5. Chi-Square may not be a valid test.			

Fisher's Exact Test	
Cell (1,1) Frequency (F)	30
Left-sided Pr <= F	0.0031
Right-sided Pr >= F	0.9998
Table Probability (P)	0.0029
Two-sided Pr <= P	0.0031

Effective Sample Size = 1145
Frequency Missing = 114

Frequency<br>Col Pct	Table of tattoo by HIV_monoinfected_vs_HIV_HCV		
	HIV_HCV	HIV_monoinfected	Total
no	29<br>90.63	986<br>86.11	1015
yes	3<br>9.38	159<br>13.89	162
Total	32	1145	1177
	Frequency Missing = 82		

Statistics for Table of tattoo by HIV_monoinfected_vs_HIV_HCV

Statistic	DF	Value	Prob
Chi-Square	1	0.5338	0.4650
Likelihood Ratio Chi-Square	1	0.5896	0.4426
Continuity Adj. Chi-Square	1	0.2214	0.6380
Mantel-Haenszel Chi-Square	1	0.5334	0.4652
Phi Coefficient		0.0213	
Contingency Coefficient		0.0213	
Cramer's V		0.0213	
WARNING: 25% of the cells have expected counts less than 5. Chi-Square may not be a valid test.			

Fisher's Exact Test	
Cell (1,1) Frequency (F)	29
Left-sided Pr <= F	0.8397
Right-sided Pr >= F	0.3373
Table Probability (P)	0.1770
Two-sided Pr <= P	0.6082

Effective Sample Size = 1177
Frequency Missing = 82

HIV_monoinfected_vs._Hepatitis

The SAS System

The FREQ Procedure

Frequency<br>Col Pct	Table of transformed_age_1 by HIV_monoinfected_vs_Hepatitis		
	**HIV_monoinfected**	**Hepatitis**	**Total**
**18-29**	476<br>40.51	26<br>32.50	502
**30-49**	505<br>42.98	42<br>52.50	547
**0-17**	55<br>4.68	2<br>2.50	57
**>_50**	139<br>11.83	10<br>12.50	149
**Total**	1175	80	1255
	**Frequency Missing = 4**		

Statistics for Table of transformed_age_1 by HIV_monoinfected_vs_Hepatitis

Statistic	DF	Value	Prob
**Chi-Square**	3	3.5721	0.3115
**Likelihood Ratio Chi-Square**	3	3.7031	0.2954
**Mantel-Haenszel Chi-Square**	1	0.4244	0.5147
**Phi Coefficient**		0.0534	
**Contingency Coefficient**		0.0533	
**Cramer's V**		0.0534	

Effective Sample Size = 1255
Frequency Missing = 4

Frequency<br>Col Pct	Table of sex by HIV_monoinfected_vs_Hepatitis		
	HIV_monoinfected	Hepatitis	Total
female	499<br>42.32	29<br>36.25	528
male	680<br>57.68	51<br>63.75	731
Total	1179	80	1259

Statistics for Table of sex by HIV_monoinfected_vs_Hepatitis

Statistic	DF	Value	Prob
Chi-Square	1	1.1351	0.2867
Likelihood Ratio Chi-Square	1	1.1511	0.2833
Continuity Adj. Chi-Square	1	0.8993	0.3430
Mantel-Haenszel Chi-Square	1	1.1342	0.2869
Phi Coefficient		0.0300	
Contingency Coefficient		0.0300	
Cramer's V		0.0300	

Fisher's Exact Test	
Cell (1,1) Frequency (F)	499
Left-sided Pr <= F	0.8820
Right-sided Pr >= F	0.1716
Table Probability (P)	0.0537
Two-sided Pr <= P	0.2952

Sample Size = 1259

Frequency<br>Col Pct	Table of marital_status by HIV_monoinfected_vs_Hepatitis		
	HIV_monoinfected	Hepatitis	Total
**divorced**	100<br>8.54	6<br>7.79	106
**married**	459<br>39.20	34<br>44.16	493
**single**	562<br>47.99	36<br>46.75	598
**widower**	50<br>4.27	1<br>1.30	51
**Total**	1171	77	1248
**Frequency Missing = 11**			

Statistics for Table of marital_status by HIV_monoinfected_vs_Hepatitis

Statistic	DF	Value	Prob
**Chi-Square**	3	2.0811	0.5557
**Likelihood Ratio Chi-Square**	3	2.6018	0.4572
**Mantel-Haenszel Chi-Square**	1	0.5966	0.4399
**Phi Coefficient**		0.0408	
**Contingency Coefficient**		0.0408	
**Cramer's V**		0.0408	

Effective Sample Size = 1248
Frequency Missing = 11

Frequency<br>Col Pct	Table of ethnicity by HIV_monoinfected_vs_Hepatitis		
	HIV_monoinfected	Hepatitis	Total
black	42<br>3.65	2<br>2.67	44
brown	400<br>34.72	28<br>37.33	428
other	6<br>0.52	0<br>0.00	6
white	704<br>61.11	45<br>60.00	749
Total	1152	75	1227

Frequency Missing = 32

Statistics for Table of ethnicity by HIV_monoinfected_vs_Hepatitis

Statistic	DF	Value	Prob
Chi-Square	3	0.7308	0.8659
Likelihood Ratio Chi-Square	3	1.1115	0.7743
Mantel-Haenszel Chi-Square	1	0.0205	0.8861
Phi Coefficient		0.0244	
Contingency Coefficient		0.0244	
Cramer's V		0.0244	

WARNING: 25% of the cells have expected counts less than 5. Chi-Square may not be a valid test.

Effective Sample Size = 1227
Frequency Missing = 32

Frequency<br>Col Pct	Table of degree_level by HIV_monoinfected_vs_Hepatitis			
		HIV_monoinfected	Hepatitis	Total
	≤ 8 years	551<br>48.72	48<br>64.00	599
	> 8 years	580<br>51.28	27<br>36.00	607
	Total	1131	75	1206
	Frequency Missing = 53			

Statistics for Table of degree_level by HIV_monoinfected_vs_Hepatitis

Statistic	DF	Value	Prob
Chi-Square	1	6.5708	0.0104
Likelihood Ratio Chi-Square	1	6.6500	0.0099
Continuity Adj. Chi-Square	1	5.9737	0.0145
Mantel-Haenszel Chi-Square	1	6.5654	0.0104
Phi Coefficient		-0.0738	
Contingency Coefficient		0.0736	
Cramer's V		-0.0738	

Fisher's Exact Test	
Cell (1,1) Frequency (F)	551
Left-sided Pr <= F	0.0070
Right-sided Pr >= F	0.9965
Table Probability (P)	0.0036
Two-sided Pr <= P	0.0120

Effective Sample Size = 1206
Frequency Missing = 53

Frequency<br>Col Pct	Table of sexual_behavior by HIV_monoinfected_vs_Hepatitis		
	**HIV_monoinfected**	**Hepatitis**	**Total**
**heterosexual**	880<br>77.13	66<br>86.84	946
**homosexual/bisexual**	261<br>22.87	10<br>13.16	271
**Total**	1141	76	1217
	**Frequency Missing = 42**		

Statistics for Table of sexual_behavior by HIV_monoinfected_vs_Hepatitis

Statistic	DF	Value	Prob
**Chi-Square**	1	3.8866	0.0487
**Likelihood Ratio Chi-Square**	1	4.3473	0.0371
**Continuity Adj. Chi-Square**	1	3.3455	0.0674
**Mantel-Haenszel Chi-Square**	1	3.8834	0.0488
**Phi Coefficient**		-0.0565	
**Contingency Coefficient**		0.0564	
**Cramer's V**		-0.0565	

Fisher's Exact Test	
**Cell (1,1) Frequency (F)**	880
**Left-sided Pr <= F**	0.0289
**Right-sided Pr >= F**	0.9868
**Table Probability (P)**	0.0156
**Two-sided Pr <= P**	0.0472

Effective Sample Size = 1217
Frequency Missing = 42

WARNING: 18% of the data are missing.

Frequency<br>Col Pct	Table of way_of_acquiring_virus by HIV_monoinfected_vs_Hepatitis		
	**HIV_monoinfected**	**Hepatitis**	**Total**
**OUTROS**	27<br>2.31	0<br>0.00	27
**sexual**	1144<br>97.69	78<br>100.00	1222
**Total**	1171	78	1249
	**Frequency Missing = 10**		

Statistics for Table of way_of_acquiring_virus by HIV_monoinfected_vs_Hepatitis

Statistic	DF	Value	Prob
Chi-Square	1	1.8382	0.1752
Likelihood Ratio Chi-Square	1	3.5217	0.0606
Continuity Adj. Chi-Square	1	0.9097	0.3402
Mantel-Haenszel Chi-Square	1	1.8367	0.1753
Phi Coefficient		0.0384	
Contingency Coefficient		0.0383	
Cramer's V		0.0384	
**WARNING: 25% of the cells have expected counts less than 5. Chi-Square may not be a valid test.**			

Fisher's Exact Test	
Cell (1,1) Frequency (F)	27
Left-sided Pr <= F	1.0000
Right-sided Pr >= F	0.1720
Table Probability (P)	0.1720
Two-sided Pr <= P	0.4072

Effective Sample Size = 1249
Frequency Missing = 10

Frequency<br>Col Pct	Table of _diagnostic_period by HIV_monoinfected_vs_Hepatitis		
	HIV_monoinfected	Hepatitis	Total
> 5 years	528<br>44.78	54<br>67.50	582
3-5 years	227<br>19.25	12<br>15.00	239
< 3 years	424<br>35.96	14<br>17.50	438
Total	1179	80	1259

Statistics for Table of transformed_diagnostic_period by HIV_monoinfected_vs_Hepatitis

Statistic	DF	Value	Prob
Chi-Square	2	16.4173	0.0003
Likelihood Ratio Chi-Square	2	17.0364	0.0002
Mantel-Haenszel Chi-Square	1	15.9250	<.0001
Phi Coefficient		0.1142	
Contingency Coefficient		0.1135	
Cramer's V		0.1142	

Sample Size = 1259

Frequency<br>Col Pct	Table of Alcohol by HIV_monoinfected_vs_Hepatitis		
	HIV_monoinfected	Hepatitis	Total
no	900<br>81.45	50<br>69.44	950
yes	205<br>18.55	22<br>30.56	227
Total	1105	72	1177

Frequency Missing = 82

Statistics for Table of Alcohol by HIV_monoinfected_vs_Hepatitis

Statistic	DF	Value	Prob
Chi-Square	1	6.2566	0.0124
Likelihood Ratio Chi-Square	1	5.5950	0.0180
Continuity Adj. Chi-Square	1	5.5093	0.0189
Mantel-Haenszel Chi-Square	1	6.2513	0.0124
Phi Coefficient		0.0729	
Contingency Coefficient		0.0727	
Cramer's V		0.0729	

Fisher's Exact Test	
Cell (1,1) Frequency (F)	900
Left-sided Pr <= F	0.9943
Right-sided Pr >= F	0.0122
Table Probability (P)	0.0065
Two-sided Pr <= P	0.0196

Effective Sample Size = 1177
Frequency Missing = 82

Frequency<br>Col Pct	Table of injectable_drugs by HIV_monoinfected_vs_Hepatitis		
	HIV_monoinfected	Hepatitis	Total
no	1104<br>99.28	68<br>95.77	1172
yes	8<br>0.72	3<br>4.23	11
Total	1112	71	1183

Frequency Missing = 76

Statistics for Table of injectable_drugs by HIV_monoinfected_vs_Hepatitis

Statistic	DF	Value	Prob
Chi-Square	1	8.9050	0.0028
Likelihood Ratio Chi-Square	1	5.0618	0.0245
Continuity Adj. Chi-Square	1	5.5058	0.0190
Mantel-Haenszel Chi-Square	1	8.8975	0.0029
Phi Coefficient		0.0868	
Contingency Coefficient		0.0864	
Cramer's V		0.0868	

WARNING: 25% of the cells have expected counts less than 5. Chi-Square may not be a valid test.

Fisher's Exact Test	
Cell (1,1) Frequency (F)	1104
Left-sided Pr <= F	0.9971
Right-sided Pr >= F	0.0241
Table Probability (P)	0.0213
Two-sided Pr <= P	0.0241

Effective Sample Size = 1183
Frequency Missing = 76

Frequency<br>Col Pct	Table of tattoo by HIV_monoinfected_vs_Hepatitis		
	HIV_monoinfected	Hepatitis	Total
no	986<br>86.11	62<br>91.18	1048
yes	159<br>13.89	6<br>8.82	165
Total	1145	68	1213

Frequency Missing = 46

Statistics for Table of tattoo by HIV_monoinfected_vs_Hepatitis

Statistic	DF	Value	Prob
Chi-Square	1	1.4000	0.2367
Likelihood Ratio Chi-Square	1	1.5601	0.2117
Continuity Adj. Chi-Square	1	1.0024	0.3167
Mantel-Haenszel Chi-Square	1	1.3989	0.2369
Phi Coefficient		-0.0340	
Contingency Coefficient		0.0340	
Cramer's V		-0.0340	

Fisher's Exact Test	
Cell (1,1) Frequency (F)	986
Left-sided Pr <= F	0.1579
Right-sided Pr >= F	0.9208
Table Probability (P)	0.0787
Two-sided Pr <= P	0.2784

Effective Sample Size = 1213
Frequency Missing = 46

The FREQ Procedure
HIV_monoinfected

HIV_monoinfected				
**HIV_monoinfected**	**Frequency**	**Percent**	**Cumulative Frequency**	**Cumulative Percent**
**HIV_monoinfected**	1179	100.00	1179	100.00

anti_HBs				
**anti_HBs**	**Frequency**	**Percent**	**Cumulative Frequency**	**Cumulative Percent**
**non_reactive**	605	53.73	605	53.73
**reagent**	521	46.27	1126	100.00

anti_HBC				
**anti_HBC**	**Frequency**	**Percent**	**Cumulative Frequency**	**Cumulative Percent**
**non_reactive**	900	83.26	900	83.26
**reagent**	181	16.74	1181	100.00

Frequency Missing = 108

The SAS System

The FREQ Procedure
HIV_HCV=reagent

HIV_HCV				
**HIV_HCV**	**Frequency**	**Percent**	**Cumulative Frequency**	**Cumulative Percent**
**reagent**	39	100.00	39	100.00

anti_HBs				
**anti_HBs**	**Frequency**	**Percent**	**Cumulative Frequency**	**Cumulative Percent**
**non_reactive**	22	68.75	22	68.75
**reagent**	10	31.25	32	100.00

anti_HBC				
**anti_HBC**	**Frequency**	**Percent**	**Cumulative Frequency**	**Cumulative Percent**
**non_reactive**	25	78.24	25	78.24
**reagent**	7	21.86	32	100.00

The SAS System

The UNIVARIATE Procedure
Variable: cd4_diagnosis (cd4_diagnosis) HIV_monoinfected_female

Moments			
**N**	476	**Sum Weights**	476
**Mean**	503.2164		
**Std Deviation**	333.5076	**Variance**	111227.3
**Skewness**	1.188964	**Kurtosis**	5.550357

Quantiles (Definition 5)	
**Quantile**	**Estimate**
**100% Max**	1636
**99%**	1636
**95%**	1115
**90%**	894
**75% Q3**	688.5
**50% Median**	450.5
**25% Q1**	265.5
**10%**	120
**5%**	62
**1%**	20

The SAS System

The UNIVARIATE Procedure
Variable: cd4_diagnosis (cd4_diagnosis) HIV_monoinfected_male

Moments			
**N**	627	**Sum Weights**	627
**Mean**	408.7177		

Moments			
**Std Deviation**	269.8876	**Variance**	72839.32
**Skewness**	1.136779	**Kurtosis**	5.630798

Quantiles (Definition 5)	
**Quantile**	**Estimate**
**100% Max**	1248
**99%**	1248
**95%**	918
**90%**	764
**75% Q3**	538
**50% Median**	379
**25% Q1**	213
**10%**	91
**5%**	43
**1%**	9

Wilcoxon Scores (Rank Sums) for Variable cd4_diagnosis (cd4_diagnosis) Classified by Variable HIV_monoinfected female vs male

female vs male	N	Sum of Scores
**female**	476	476
**male**	627	627

Wilcoxon Two-Sample Test	
**Statistic**	
**U**	124944.00
**Z**	4.6340
**One-Sided Pr < Z**	<0.0001
**Two-Sided Pr > |Z|**	<0.0001

The SAS System

The UNIVARIATE Procedure

Variable: cd4_diagnosis (cd4_diagnosis) HIV_HBV_female

Moments			
**N**	16	**Sum Weights**	16
**Mean**	504.6875		
**Std Deviation**	223.0048	**Variance**	49731.16
**Skewness**	1.231659	**Kurtosis**	5.26303

Quantiles (Definition 5)	
**Quantile**	**Estimate**
**100% Max**	1138
**99%**	1138
**95%**	1138
**90%**	718
**75% Q3**	590.5
**50% Median**	475
**25% Q1**	383.5
**10%**	257
**5%**	161
**1%**	161

The SAS System

The UNIVARIATE Procedure
Variable: cd4_diagnosis (cd4_diagnosis) HIV_HBV_male

Moments			
**N**	21	**Sum Weights**	21
**Mean**	376.1905		
**Std Deviation**	214.5259	**Variance**	46021.36
**Skewness**	0.7132155	**Kurtosis**	3.619756

Quantiles (Definition 5)	
**Quantile**	**Estimate**
**100% Max**	938
**99%**	938
**95%**	701
**90%**	638
**75% Q3**	477
**50% Median**	336

Quantiles (Definition 5)	
**Quantile**	**Estimate**
**25% Q1**	258
**10%**	156
**5%**	74
**1%**	17

Wilcoxon Scores (Rank Sums) for Variable cd4_diagnosis (cd4_diagnosis) Classified by Variable HIV_HBV female vs male		
**female vs male**	**N**	**Sum of Scores**
**Female**	16	16
**male**	21	21

Wilcoxon Two-Sample Test	
**Statistic**	
**U**	108.00
**Z**	1.8394
**One-Sided Pr < Z**	0.0329
**Two-Sided Pr > |Z|**	0.0659

The SAS System

The UNIVARIATE Procedure

Variable: cd4_diagnosis (cd4_diagnosis) HIV_HCV_female

Moments			
**N**	11	**Sum Weights**	11
**Mean**	360.2727		
**Std Deviation**	184.5487	**Variance**	34058.22
**Skewness**	0.1671016	**Kurtosis**	1.675045

Quantiles (Definition 5)	
**Quantile**	**Estimate**
**100% Max**	666
**99%**	666
**95%**	666

Quantiles (Definition 5)	
**Quantile**	**Estimate**
**90%**	568
**75% Q3**	506
**50% Median**	410
**25% Q1**	185
**10%**	141
**5%**	134
**1%**	134

The SAS System

The UNIVARIATE Procedure

Variable: cd4_diagnosis (cd4_diagnosis) HIV_HCV_male

Moments			
**N**	25	**Sum Weights**	25
**Mean**	421.15		
**Std Deviation**	249.9246	**Variance**	62462.31
**Skewness**	1.359291	**Kurtosis**	5.100264

Quantiles (Definition 5)	
**Quantile**	**Estimate**
**100% Max**	1211
**99%**	1211
**95%**	815
**90%**	726
**75% Q3**	522
**50% Median**	345
**25% Q1**	243
**10%**	154
**5%**	147
**1%**	98

Wilcoxon Scores (Rank Sums) for Variable cd4_diagnosis (cd4_diagnosis) Classified by Variable HIV_HCV female vs male		
**female vs male**	**N**	**Sum of Scores**
**female**	11	11
**male**	25	25

Wilcoxon Two-Sample Test	
**Statistic**	
**U**	119
**Z**	0.6353
**One-Sided Pr < Z**	0.2626
**Two-Sided Pr > |Z|**	0.5252

The SAS System

The UNIVARIATE Procedure

Variable: cd4_diagnosis (cd4_diagnosis) All_cd4_≤ 18_age

Moments			
**N**	71	**Sum Weights**	71
**Mean**	660.0423		
**Std Deviation**	379.138	**Variance**	143745.6
**Skewness**	1.312528	**Kurtosis**	4.967981

Quantiles (Definition 5)	
**Quantile**	**Estimate**
**100% Max**	2001
**99%**	2001
**95%**	1402
**90%**	1175
**75% Q3**	764
**50% Median**	582
**25% Q1**	423
**10%**	265
**5%**	156
**1%**	97

The SAS System

The UNIVARIATE Procedure

Variable: cd4_diagnosis (cd4_diagnosis) All_cd4_≥_50_age

Moments			
**N**	139	**Sum Weights**	139
**Mean**	374.3094		
**Std Deviation**	263.7228	**Variance**	69549.71
**Skewness**	1.321624	**Kurtosis**	5.486856

Quantiles (Definition 5)	
**Quantile**	**Estimate**
**100% Max**	1361
**99%**	1361
**95%**	838
**90%**	756
**75% Q3**	507
**50% Median**	333
**25% Q1**	168
**10%**	97
**5%**	54
**1%**	20

Wilcoxon Scores (Rank Sums) for Variable cd4_diagnosis (cd4_diagnosis) Classified by Variable All_≤ 18_age_vs _≥ 50_age

≤ 18_age_vs _>50_age	N	Sum of Scores
**≤ 18_age**	71	71
**≥ 50_age**	139	139

Wilcoxon Two-Sample Test	
**Statistic**	
**U**	2447.50
**Z**	5.9702
**One-Sided Pr < Z**	<0.0001
**Two-Sided Pr > |Z|**	<0.0001

Table 3. CD4+ T-cell count and HIV viral load and their association with HBV and HCV in HIV-infected patients at baseline

The SAS System

Frequency<br>Col Pct	Table of cd4_diagnosis_transformed by HIV_monoinfected_vs_HIV_HBV		
	**HIV_HBV**	**HIV_monoinfected**	**Total**
**200-500**	20<br>54.06	473<br>42.88	493
**<200**	5<br>13.51	232<br>21.03	237
**>500**	12<br>32.43	398<br>36.08	410
**Total**	37	1103	1140
	**Frequency Missing = 119**		

Statistics for Table of cd4_diagnosis_transformed by HIV_monoinfected_vs_HIV_HBV

Statistic	DF	Value	Prob
**Chi-Square**	2	2.1395	0.3371
**Likelihood Ratio Chi-Square**	2	2.2042	0.3322
**Mantel-Haenszel Chi-Square**	1	0.9987	0.3176
**Phi Coefficient**		0.0433	
**Contingency Coefficient**		0.0433	
**Cramer's V**		0.0433	

Effective Sample Size = 1140
Frequency Missing = 119

Frequency<br>Col Pct	Table of cd4_diagnosis_transformed by HIV_monoinfected_vs_HIV_HCV		
	HIV_HCV	HIV_monoinfected	Total
**200-500**	20<br>55.55	473<br>42.88	493
**<200**	6<br>16.67	232<br>21.03	238
**>500**	10<br>27.78	398<br>36.08	408
**Total**	36	1103	1139

Frequency Missing = 120

Statistics for Table of cd4_diagnosis_transformed by HIV_monoinfected_vs_HIV_HCV

Statistic	DF	Value	Prob
**Chi-Square**	2	2.2830	0.3143
**Likelihood Ratio Chi-Square**	2	2.2572	0.3235
**Mantel-Haenszel Chi-Square**	1	1.9515	0.1624
**Phi Coefficient**		0.0448	
**Contingency Coefficient**		0.0447	
**Cramer's V**		0.0448	

Effective Sample Size = 1139
Frequency Missing = 120

Frequency<br>Col Pct	Table of cd4_diagnosis_transformed by HIV_monoinfected_vs_Hepatitis		
	HIV_monoinfected	Hepatitis	Total
**200-500**	473	42	515
	42.88	56.00	
**<200**	232	11	243
	21.03	14.67	
**>500**	398	22	420
	36.08	29.33	
**Total**	1103	75	1178

Frequency Missing = 81

Statistics for Table of cd4_diagnosis_transformed by HIV_monoinfected_vs_Hepatitis

Statistic	DF	Value	Prob
**Chi-Square**	2	5.0412	0.0774
**Likelihood Ratio Chi-Square**	2	5.0293	0.0809
**Mantel-Haenszel Chi-Square**	1	3.5180	0.0607
**Phi Coefficient**		0.0654	
**Contingency Coefficient**		0.0653	
**Cramer's V**		0.0654	

Effective Sample Size = 1178
Frequency Missing = 81

Frequency<br>Col Pct	Table of cd4_diagnosis_transformed by HIV_HBV_vs_HIV_HCV		
	**HIV_HBV**	**HIV_HCV**	**Total**
**200-500**	5<br>13.51	6<br>16.67	11
**<200**	20<br>54.05	20<br>55.56	40
**>500**	12<br>32.43	10<br>27.78	22
**Total**	37	36	73

Statistics for Table of cd4_diagnosis_transformed by HIV_HBV_vs_HIV_HCV

Statistic	DF	Value	Prob
**Chi-Square**	2	2.2591	0.8790
**Likelihood Ratio Chi-Square**	2	2.0293	0.8809
**Mantel-Haenszel Chi-Square**	1	2.5180	0.8607
**Phi Coefficient**		0.0234	
**Contingency Coefficient**		0.0356	
**Cramer's V**		0.0346	

The UNIVARIATE Procedure
Variable: cd4_diagnosis (cd4_diagnosis)
HIV_monoinfected

Moments			
**N**	1103	**Sum Weights**	1103
**Mean**	449.499547		
**Std Deviation**	302.509462	**Variance**	91511.9744
**Skewness**	1.24370692	**Kurtosis**	2.96352292

Quantiles (Definition 5)	
**Quantile**	**Estimate**
**100% Max**	2283
**99%**	1408
**95%**	974
**90%**	828
**75% Q3**	608
**50% Median**	405
**25% Q1**	234
**10%**	99
**5%**	54
**1%**	14
**0% Min**	2

The SAS System

The UNIVARIATE Procedure
Variable: cd4_diagnosis (cd4_diagnosis) HIV_HBV

Moments			
**N**	37	**Sum Weights**	37
**Mean**	431.756757		
**Std Deviation**	224.619234	**Variance**	50453.8003
**Skewness**	0.90223774	**Kurtosis**	1.86296389

Quantiles (Definition 5)	
**Quantile**	**Estimate**
**100% Max**	1138
**99%**	1138
**95%**	938
**90%**	701
**75% Q3**	557
**50% Median**	387
**25% Q1**	287
**10%**	161
**5%**	74
**1%**	17

The NPAR1WAY Procedure

Wilcoxon Scores (Rank Sums) for Variable cd4_diagnosis Classified by Variable HIV_monoinfected_vs_HIV_HBV		
**HIV_monoinfected_vs_HIV_HBV**	**N**	**Sum of Scores**
**HIV_monoinfected**	1103	629075.0
**HIV_HBV**	37	21295.0
**Average scores were used for ties.**		

Wilcoxon Two-Sample Test	
**Statistic**	21295.0000
**Normal Approximation**	
**Z**	0.0944
**One-Sided Pr > Z**	0.4624

Wilcoxon Two-Sample Test	
**Two-Sided Pr > |Z|**	0.9248

The UNIVARIATE Procedure
Variable: cd4_diagnosis (cd4_diagnosis) HIV_HCV

Moments			
**N**	36	**Sum Weights**	36
**Mean**	402.5556		
**Std Deviation**	231.0224	**Variance**	53371.34
**Skewness**	1.289724	**Kurtosis**	5.348068

Quantiles (Definition 5)	
**Quantile**	**Estimate**
**100% Max**	1211
**99%**	1211
**95%**	815
**90%**	697
**75% Q3**	514
**50% Median**	377.5
**25% Q1**	228.5
**10%**	147
**5%**	134
**1%**	98

The SAS System

The NPAR1WAY Procedure

Wilcoxon Scores (Rank Sums) for Variable cd4_diagnosis Classified by Variable HIV_monoinfected_vs_HIV_HCV

HIV_monoinfected_vs_HIV_HCV	N	Sum of Scores
**HIV_monoinfected**	1103	630146
**HIV_HCV**	36	19084

Average scores were used for ties.

Wilcoxon Two-Sample Test	
**Statistic**	18418.000
**Normal Approximation**	

Wilcoxon Two-Sample Test	
**Z**	0.7394
**One-Sided Pr > Z**	0.2298
**Two-Sided Pr > |Z|**	0.4597

The UNIVARIATE Procedure
Variable: cd4_diagnosis (cd4_diagnosis) HIV_hepatitis

Moments			
**N**	75	**Sum Weights**	75
**Mean**	417.3733		
**Std Deviation**	223.6863	**Variance**	50035.56
**Skewness**	1.084653	**Kurtosis**	4.970194

Quantiles (Definition 5)	
**Quantile**	**Estimate**
**100% Max**	1211
**99%**	1211
**95%**	815
**90%**	697
**75% Q3**	522
**50% Median**	387
**25% Q1**	257
**10%**	156
**5%**	134
**1%**	17

The NPAR1WAY Procedure

Wilcoxon Scores (Rank Sums) for Variable cd4_diagnosis Classified by Variable HIV_monoinfected_vs_HIV_hepatitis

HIV_monoinfected_vs_HIV_HCV	N	Sum of Scores
**HIV_monoinfected**	1103	651430.5
**HIV_hepatitis**	75	43000.5

Average scores were used for ties.

Wilcoxon Two-Sample Test	
**Statistic**	40150.50

Wilcoxon Two-Sample Test	
**Normal Approximation**	
**Z**	0.4251
**One-Sided Pr > Z**	0.3354
**Two-Sided Pr > |Z|**	0.6707

The NPAR1WAY Procedure

Wilcoxon Scores (Rank Sums) for Variable cd4_diagnosis Classified by Variable HIV_HBV_vs_HIV_HCV		
**HIV_HBV_vs_HIV_HCV**	**N**	**Sum of Scores**
**HIV_HBV**	37	1452.5
**HIV_HCV**	36	1248.5
**Average scores were used for ties.**		

Wilcoxon Two-Sample Test	
**Statistic**	582.50
**Normal Approximation**	
**Z**	0.9213
**One-Sided Pr > Z**	0.1784
**Two-Sided Pr > |Z|**	0.3569

The UNIVARIATE Procedure
Variable: HIV_viral_load_LOG (HIV_viral_load_LOG)
HIV_monoinfected_

Moments			
**N**	1098	**Sum Weights**	1098
**Mean**	4.02112754		
**Std Deviation**	0.61235927	**Variance**	0.37498388
**Skewness**	-0.7940259	**Kurtosis**	0.56704082

Quantiles (Definition 5)	
**Quantile**	**Estimate**
**100% Max**	4.99397
**99%**	4.96757
**95%**	4.87880
**90%**	4.75727
**75% Q3**	4.44988
**50% Median**	4.09915
**25% Q1**	3.64205
**10%**	3.11926
**5%**	2.85673
**1%**	2.26007
**0% Min**	1.66745

The UNIVARIATE Procedure
Variable: HIV_viral_load_LOG (HIV_viral_load_LOG)
HIV_monoinfected_vs_HIV_HBV=HIV_HBV

Moments			
**N**	34	**Sum Weights**	34
**Mean**	3.80586445		
**Std Deviation**	1.09463913	**Variance**	1.19823482
**Skewness**	-0.69925	**Kurtosis**	-0.4538951

Quantiles (Definition 5)	
**Quantile**	**Estimate**
**100% Max**	5.32314
**99%**	5.32314
**95%**	5.18959
**90%**	5.08189
**75% Q3**	4.49868
**50% Median**	4.11798
**25% Q1**	3.17026
**10%**	1.86332
**5%**	1.60206
**1%**	1.60206
**0% Min**	1.60206

Wilcoxon Scores (Rank Sums) for Variable HIV_viral_load_LOG Classified by Variable HIV_monoinfected_vs_HIV_HBV

HIV_monoinfected_vs_HIV_HBV	N	Sum of Scores
**HIV_monoinfected**	1098	622804.0
**HIV_HBV**	34	18474.0

Wilcoxon Two-Sample Test	
**Statistic**	18474.0000
**Z**	-0.4189
**One-Sided Pr < Z**	0.3376
**Two-Sided Pr > |Z|**	0.6753

The SAS System

The UNIVARIATE Procedure
Variable: HIV_viral_load_LOG_HIV_HCV

Moments			
**N**	27	**Sum Weights**	27
**Mean**	4.236667		
**Std Deviation**	.9510237	**Variance**	.904446
**Skewness**	-.5194416	**Kurtosis**	3.514769

Quantiles (Definition 5)	
**Quantile**	**Estimate**
**100% Max**	5.95
**99%**	5.95
**95%**	5.68
**90%**	5.68
**75% Q3**	4.81
**50% Median**	4.24
**25% Q1**	3.77
**10%**	2.83
**5%**	2.65
**1%**	1.7

Wilcoxon Scores (Rank Sums) for Variable HIV_viral_load_LOG Classified by Variable HIV_monoinfected_vs_HIV_HCV

HIV_monoinfected_vs_HIV_HCV	N	Sum of Scores
**HIV_monoinfected**	1098	615581.5
**HIV_HCV**	27	17793.5

Wilcoxon Two-Sample Test	
**Statistic**	12230.50
**Normal Approximation**	
**Z**	1.5544
**One-Sided Pr < Z**	0.0600
**Two-Sided Pr > |Z|**	0.1201

The SAS System

The UNIVARIATE Procedure

Variable: HIV_viral_load_LOG HIV_hepatitis

Moments			
**N**	63	**Sum Weights**	63
**Mean**	3.984444		
**Std Deviation**	1.042074	**Variance**	1.085919
**Skewness**	0.6399357	**Kurtosis**	2.940407

Quantiles (Definition 5)	
**Quantile**	**Estimate**
**100% Max**	5.95
**99%**	5.95
**95%**	5.32
**90%**	5.14
**75% Q3**	4.70
**50% Median**	4.12
**25% Q1**	3.47
**10%**	2.65
**5%**	1.72
**1%**	1.6

Wilcoxon Scores (Rank Sums) for Variable HIV_viral_load_LOG Classified by Variable HIV_monoinfected_vs_HIV_hepatitis

HIV_monoinfected_vs_HIV_hepatitis	N	Sum of Scores
**HIV_monoinfected**	1098	636424
**HIV_hepatitis**	63	38117

Wilcoxon Two-Sample Test	
**Statistic**	33073
**Normal Approximation**	
**Z**	0.5850
**One-Sided Pr < Z**	0.2793
**Two-Sided Pr > |Z|**	0.5586

Wilcoxon Scores (Rank Sums) for Variable HIV_viral_load_LOG Classified by Variable HIV_HBV_vs_HIV_HCV		
**HIV_HBV_vs_HIV_HCV**	**N**	**Sum of Scores**
**HIV_HBV**	30	777.0
**HIV_HCV**	27	876.0

Wilcoxon Two-Sample Test	
**Statistic**	312.00
**Normal Approximation**	
**Z**	14863
**One-Sided Pr < Z**	0.0686
**Two-Sided Pr > |Z|**	0.1372

Table 4. Effect of cART on CD4+ T-cell count, CD4/CD8 ratio and HIV viral load in HIV-coinfected patients

The SAS System

The UNIVARIATE Procedure
Variable: HBV (HBV)
cd_4_naïve

Moments			
**N**	24	**Sum Weights**	24
**Mean**	347.541667	**Sum Observations**	8341
**Std Deviation**	198.75569	**Variance**	39503.8243
**Skewness**	0.83695443	**Kurtosis**	0.84732151
**Uncorrected SS**	3807433	**Corrected SS**	908587.958
**Coeff Variation**	57.1890248	**Std Error Mean**	40.5708353

Quantiles (Definition 5)	
**Quantile**	**Estimate**
**100% Max**	869.0
**99%**	869.0
**95%**	701.0
**90%**	595.0
**75% Q3**	465.5
**50% Median**	316.0
**25% Q1**	226.5
**10%**	128.0
**5%**	74.0
**1%**	30.0
**0% Min**	30.0

The SAS System

The UNIVARIATE Procedure
Variable: HBV (HBV)
Treat=cd_4_time_12_months

Moments			
**N**	24	**Sum Weights**	24
**Mean**	514.625	**Sum Observations**	12351
**Std Deviation**	230.574861	**Variance**	53164.7663
**Skewness**	1.16483499	**Kurtosis**	1.96882452
**Uncorrected SS**	7578923	**Corrected SS**	1222789.63
**Coeff Variation**	44.8044422	**Std Error Mean**	47.0658963

Quantiles (Definition 5)	
**Quantile**	**Estimate**
**100% Max**	1103.0
**99%**	1103.0
**95%**	1101.0
**90%**	678.0
**75% Q3**	612.5
**50% Median**	512.0
**25% Q1**	346.0
**10%**	246.0
**5%**	243.0
**1%**	194.0
**0% Min**	194.0

The SAS System
The NPAR1WAY Procedure
Treat=cd_4 count

Wilcoxon Scores (Rank Sums) for Variable HBV Classified by Variable time_0_vs_12

time_0_vs_12	N	Sum of Scores	Expected Under H0	Std Dev Under H0	Mean Score
**0**	24	462.0	588.0	48.497423	19.250
**12**	24	714.0	588.0	48.497423	29.750

Wilcoxon Two-Sample Test

**Statistic**	462.0000
**Normal Approximation**	
**Z**	-2.5878
**One-Sided Pr < Z**	0.0048
**Two-Sided Pr > |Z|**	0.0097
**t Approximation**	
**One-Sided Pr < Z**	0.0064
**Two-Sided Pr > |Z|**	0.0128

Z includes a continuity correction of 0.5.

Kruskal-Wallis Test

**Chi-Square**	6.7500
**DF**	1
**Pr > Chi-Square**	0.0094

The SAS System

The UNIVARIATE Procedure
Variable: HBV (HBV)
Treat=cd_4_time_24_months

Moments			
**N**	24	**Sum Weights**	24
**Mean**	597.5	**Sum Observations**	14340
**Std Deviation**	229.043759	**Variance**	52461.0435
**Skewness**	0.13367518	**Kurtosis**	-0.0836305
**Uncorrected SS**	9774754	**Corrected SS**	1206604
**Coeff Variation**	38.3336835	**Std Error Mean**	46.7533615

Quantiles (Definition 5)	
**Quantile**	**Estimate**
**100% Max**	1127.0
**99%**	1127.0
**95%**	908.0
**90%**	835.0
**75% Q3**	747.5
**50% Median**	613.0
**25% Q1**	423.0
**10%**	312.0
**5%**	280.0
**1%**	170.0
**0% Min**	170.0

The NPAR1WAY Procedure
Treat=cd_4 cell count

Wilcoxon Scores (Rank Sums) for Variable HBV Classified by Variable time_0_vs_24

time_0_vs_24	N	Sum of Scores	Expected Under H0	Std Dev Under H0	Mean Score
0	24	414.50	588.0	48.494790	17.270833
24	24	761.50	588.0	48.494790	31.729167

Average scores were used for ties.

Wilcoxon Two-Sample Test

**Statistic**	414.5000
**Normal Approximation**	
**Z**	-3.5674
**One-Sided Pr < Z**	0.0002
**Two-Sided Pr > |Z|**	0.0004
**t Approximation**	
**One-Sided Pr < Z**	0.0004
**Two-Sided Pr > |Z|**	0.0008

Z includes a continuity correction of 0.5.

Kruskal-Wallis Test

**Chi-Square**	12.8000
**DF**	1
**Pr > Chi-Square**	0.0003

The NPAR1WAY Procedure
Treat=cd_4_count

Wilcoxon Scores (Rank Sums) for Variable HBV Classified by Variable time_12_vs_24

time_12_vs_24	N	Sum of Scores	Expected Under H0	Std Dev Under H0	Mean Score
12	24	503.50	588.0	48.494790	20.979167
24	24	672.50	588.0	48.494790	28.020833

Average scores were used for ties.

Wilcoxon Two-Sample Test

**Statistic**	503.5000
**Normal Approximation**	
**Z**	-1.7321
**One-Sided Pr < Z**	0.0416
**Two-Sided Pr > |Z|**	0.0832
**t Approximation**	
**One-Sided Pr < Z**	0.0449
**Two-Sided Pr > |Z|**	0.0898

Z includes a continuity correction of 0.5.

Kruskal-Wallis Test

**Chi-Square**	3.0362
**DF**	1
**Pr > Chi-Square**	0.0814

The SAS System

The UNIVARIATE Procedure
Variable: HBV (HBV)
cd_4:cd_8_naïve

Moments			
**N**	24	**Sum Weights**	24
**Mean**	0.40958333	**Sum Observations**	9.83
**Std Deviation**	0.35201876	**Variance**	0.12391721
**Skewness**	1.92805077	**Kurtosis**	4.49438498
**Uncorrected SS**	6.8763	**Corrected SS**	2.85009583
**Coeff Variation**	85.9455782	**Std Error Mean**	0.07185553

Quantiles (Definition 5)	
**Quantile**	**Estimate**
**100% Max**	1.590
**99%**	1.590
**95%**	0.960
**90%**	0.900
**75% Q3**	0.585
**50% Median**	0.295
**25% Q1**	0.185
**10%**	0.120
**5%**	0.080
**1%**	0.040
**0% Min**	0.040

The SAS System

The UNIVARIATE Procedure
Variable: HBV (HBV)
Treat=cd_4:cd_8 time_12

Moments			
**N**	24	**Sum Weights**	24
**Mean**	0.58375	**Sum Observations**	14.01
**Std Deviation**	0.40338581	**Variance**	0.16272011
**Skewness**	1.60015192	**Kurtosis**	2.77777703
**Uncorrected SS**	11.9209	**Corrected SS**	3.7425625
**Coeff Variation**	69.1024936	**Std Error Mean**	0.08234078

Quantiles (Definition 5)	
**Quantile**	**Estimate**
**100% Max**	1.81
**99%**	1.81
**95%**	1.39
**90%**	1.08
**75% Q3**	0.70
**50% Median**	0.48
**25% Q1**	0.30
**10%**	0.20
**5%**	0.19
**1%**	0.11
**0% Min**	0.11

The NPAR1WAY Procedure
Treat=cd_4/cd_8

Wilcoxon Scores (Rank Sums) for Variable HBV Classified by Variable time_0_vs_12

time_0_vs_12	N	Sum of Scores	Expected Under H0	Std Dev Under H0	Mean Score
**0**	24	491.50	588.0	48.486892	20.479167
**12**	24	684.50	588.0	48.486892	28.520833

Average scores were used for ties.

Wilcoxon Two-Sample Test	
**Statistic**	491.5000
**Normal Approximation**	
**Z**	-1.9799
**One-Sided Pr < Z**	0.0239
**Two-Sided Pr > |Z|**	0.0477
**t Approximation**	
**One-Sided Pr < Z**	0.0268
**Two-Sided Pr > |Z|**	0.0536

Z includes a continuity correction of 0.5.

Kruskal-Wallis Test	
**Chi-Square**	3.9610
**DF**	1
**Pr > Chi-Square**	0.0466

The UNIVARIATE Procedure
Variable: HBV (HBV)
Treat=cd_4:cd_8 time_24_months

Moments			
**N**	24	**Sum Weights**	24
**Mean**	0.71291667	**Sum Observations**	17.11
**Std Deviation**	0.35799537	**Variance**	0.12816069
**Skewness**	1.03779198	**Kurtosis**	1.85730225
**Uncorrected SS**	15.1457	**Corrected SS**	2.94769583
**Coeff Variation**	50.215599	**Std Error Mean**	0.0730755

Quantiles (Definition 5)	
**Quantile**	**Estimate**
**100% Max**	1.740
**99%**	1.740
**95%**	1.390
**90%**	0.970
**75% Q3**	0.885
**50% Median**	0.655
**25% Q1**	0.480
**10%**	0.330
**5%**	0.230
**1%**	0.170
**0% Min**	0.170

The NPAR1WAY Procedure
Treat=cd_4/cd_8

Wilcoxon Scores (Rank Sums) for Variable HBV Classified by Variable time_0_vs_24

time_0_24	N	Sum of Scores	Expected Under H0	Std Dev Under H0	Mean Score
**0**	24	430.0	588.0	48.481626	17.916667
**24**	24	746.0	588.0	48.481626	31.083333

Average scores were used for ties.

Wilcoxon Two-Sample Test

**Statistic**	430.0000
**Normal Approximation**	
**Z**	-3.2487
**One-Sided Pr < Z**	0.0006
**Two-Sided Pr > |Z|**	0.0012
**t Approximation**	
**One-Sided Pr < Z**	0.0011
**Two-Sided Pr > |Z|**	0.0021

Z includes a continuity correction of 0.5.

Kruskal-Wallis Test

**Chi-Square**	10.6209
**DF**	1
**Pr > Chi-Square**	0.0011

The NPAR1WAY Procedure
Treat=cd_4/cd_8

Wilcoxon Scores (Rank Sums) for Variable HBV Classified by Variable time_12_vs_24

time_12_vs_24	N	Sum of Scores	Expected Under H0	Std Dev Under H0	Mean Score
**12**	24	508.0	588.0	48.481626	21.166667
**24**	24	668.0	588.0	48.481626	27.833333

Average scores were used for ties.

Wilcoxon Two-Sample Test

**Statistic**	508.0000
**Normal Approximation**	
**Z**	-1.6398
**One-Sided Pr < Z**	0.0505
**Two-Sided Pr > |Z|**	0.1010
**t Approximation**	
**One-Sided Pr < Z**	0.0539
**Two-Sided Pr > |Z|**	0.1077

Z includes a continuity correction of 0.5.

Kruskal-Wallis Test

**Chi-Square**	2.7229
**DF**	1
**Pr > Chi-Square**	0.0989

The SAS System

The UNIVARIATE Procedure
Variable: HBV (HBV)
cv_log_naïve

Moments			
**N**	20	**Sum Weights**	20
**Mean**	3.90788235	**Sum Observations**	78.157647
**Std Deviation**	1.38726974	**Variance**	1.92451734
**Skewness**	-0.643462	**Kurtosis**	-1.0758625
**Uncorrected SS**	341.996718	**Corrected SS**	36.5658295
**Coeff Variation**	35.499271	**Std Error Mean**	0.31020295

Quantiles (Definition 5)	
**Quantile**	**Estimate**
**100% Max**	5.49958
**99%**	5.49958
**95%**	5.46713
**90%**	5.37892
**75% Q3**	5.10267
**50% Median**	4.25391
**25% Q1**	2.64960
**10%**	1.69897
**5%**	1.69897
**1%**	1.69897
**0% Min**	1.69897

The UNIVARIATE Procedure
Variable: HBV (HBV)
Treat=cv_log time_12_months

Moments			
**N**	24	**Sum Weights**	24
**Mean**	1.84097179	**Sum Observations**	44.183323
**Std Deviation**	0.36142809	**Variance**	0.13063026
**Skewness**	1.39137635	**Kurtosis**	2.04905513
**Uncorrected SS**	84.3447474	**Corrected SS**	3.00449606
**Coeff Variation**	19.6324621	**Std Error Mean**	0.0737762

Quantiles (Definition 5)	
**Quantile**	**Estimate**
**100% Max**	2.80889
**99%**	2.80889
**95%**	2.60206
**90%**	2.55630
**75% Q3**	1.90224
**50% Median**	1.69897
**25% Q1**	1.69897
**10%**	1.65321
**5%**	1.30103
**1%**	1.30103
**0% Min**	1.30103

The NPAR1WAY Procedure
Treat=cv_log

Wilcoxon Scores (Rank Sums) for Variable HBV Classified by Variable time_0_vs_12

time_0_vs_12	N	Sum of Scores	Expected Under H0	Std Dev Under H0	Mean Score
**0**	20	627.0	450.0	41.739148	31.3500
**12**	24	363.0	540.0	41.739148	15.1250

Average scores were used for ties.

Wilcoxon Two-Sample Test

**Statistic**	627.0000
**Normal Approximation**	
**Z**	4.2286
**One-Sided Pr > Z**	<.0001
**Two-Sided Pr > |Z|**	<.0001
**t Approximation**	
**One-Sided Pr > Z**	<.0001
**Two-Sided Pr > |Z|**	0.0001

Z includes a continuity correction of 0.5.

Kruskal-Wallis Test

**Chi-Square**	17.9829
**DF**	1
**Pr > Chi-Square**	<.0001

The UNIVARIATE Procedure
Variable: HBV (HBV)
Treat=cv_log time_24_months

Moments			
**N**	24	**Sum Weights**	24
**Mean**	1.64018853	**Sum Observations**	39.3645247
**Std Deviation**	0.34071471	**Variance**	0.11608651
**Skewness**	2.68138938	**Kurtosis**	10.8251556
**Uncorrected SS**	67.2352315	**Corrected SS**	2.6699898
**Coeff Variation**	20.7728992	**Std Error Mean**	0.0695481

Quantiles (Definition 5)	
**Quantile**	**Estimate**
**100% Max**	2.99056
**99%**	2.99056
**95%**	1.83251
**90%**	1.75587
**75% Q3**	1.69897
**50% Median**	1.69897
**25% Q1**	1.39794
**10%**	1.30103
**5%**	1.30103
**1%**	1.30103
**0% Min**	1.30103

The NPAR1WAY Procedure
Treat=cv_log

Wilcoxon Scores (Rank Sums) for Variable HBV Classified by Variable time_0_vs_24

time_0_vs_24	N	Sum of Scores	Expected Under H0	Std Dev Under H0	Mean Score
0	20	650.0	450.0	41.151502	32.500000
24	24	340.0	540.0	41.151502	14.166667

Average scores were used for ties.

Wilcoxon Two-Sample Test

Statistic	650.0000
Normal Approximation	
Z	4.8479
One-Sided Pr > Z	<.0001
Two-Sided Pr > |Z|	<.0001
t Approximation	
One-Sided Pr > Z	<.0001
Two-Sided Pr > |Z|	<.0001

Z includes a continuity correction of 0.5.

Kruskal-Wallis Test

Chi-Square	23.6205
DF	1
Pr > Chi-Square	<.0001

The NPAR1WAY Procedure
Treat=cv_log

Wilcoxon Scores (Rank Sums) for Variable HBV Classified by Variable time_12_vs_24

time_12_vs_24	N	Sum of Scores	Expected Under H0	Std Dev Under H0	Mean Score
**12**	24	692.0	588.0	45.672283	28.833333
**24**	24	484.0	588.0	45.672283	20.166667

Average scores were used for ties.

Wilcoxon Two-Sample Test

**Statistic**	692.0000
**Normal Approximation**	
**Z**	2.2661
**One-Sided Pr > Z**	0.0117
**Two-Sided Pr > |Z|**	0.0234
**t Approximation**	
**One-Sided Pr > Z**	0.0140
**Two-Sided Pr > |Z|**	0.0281

Z includes a continuity correction of 0.5.

Kruskal-Wallis Test

**Chi-Square**	5.1851
**DF**	1
**Pr > Chi-Square**	0.0228

The SAS System

The UNIVARIATE Procedure
Variable: HCV (HCV)
cd_4_naïve

Moments			
**N**	23	**Sum Weights**	23
**Mean**	364.521739	**Sum Observations**	8384
**Std Deviation**	269.03884	**Variance**	72381.8972
**Skewness**	1.82338367	**Kurtosis**	3.35485997
**Uncorrected SS**	4648552	**Corrected SS**	1592401.74
**Coeff Variation**	73.8059794	**Std Error Mean**	56.098476

Quantiles (Definition 5)	
**Quantile**	**Estimate**
**100% Max**	1171
**99%**	1171
**95%**	977
**90%**	697
**75% Q3**	478
**50% Median**	304
**25% Q1**	154
**10%**	141
**5%**	140
**1%**	67
**0% Min**	67

The SAS System

The UNIVARIATE Procedure
Variable: HCV (HCV)
Treat=cd_4 time_12_months

Moments			
**N**	23	**Sum Weights**	23
**Mean**	407.934633	**Sum Observations**	9382.49657
**Std Deviation**	208.678839	**Variance**	43546.8577
**Skewness**	1.43441038	**Kurtosis**	1.88947398
**Uncorrected SS**	4785476.17	**Corrected SS**	958030.87
**Coeff Variation**	51.1549699	**Std Error Mean**	43.5125458

Quantiles (Definition 5)	
**Quantile**	**Estimate**
**100% Max**	1009.33
**99%**	1009.33
**95%**	786.00
**90%**	706.00
**75% Q3**	555.50
**50% Median**	344.00
**25% Q1**	252.00
**10%**	209.00
**5%**	208.00
**1%**	191.00
**0% Min**	191.00

The NPAR1WAY Procedure
Treat=cd_4

Wilcoxon Scores (Rank Sums) for Variable HCV Classified by Variable time_0_vs_12

time_0_vs_12	N	Sum of Scores	Expected Under H0	Std Dev Under H0	Mean Score
0	23	477.50	540.50	45.516908	20.760870
12	23	603.50	540.50	45.516908	26.239130

Average scores were used for ties.

Wilcoxon Two-Sample Test

**Statistic**	477.5000
**Normal Approximation**	
**Z**	-1.3731
**One-Sided Pr < Z**	0.0849
**Two-Sided Pr > |Z|**	0.1697
**t Approximation**	
**One-Sided Pr < Z**	0.0883
**Two-Sided Pr > |Z|**	0.1765

Z includes a continuity correction of 0.5.

Kruskal-Wallis Test

**Chi-Square**	1.9157
**DF**	1
**Pr > Chi-Square**	0.1663

The SAS System

The UNIVARIATE Procedure
Variable: HCV (HCV)
Treat=cd_4 time_24_months

Moments			
**N**	23	**Sum Weights**	23
**Mean**	521.094893	**Sum Observations**	11985.1825
**Std Deviation**	179.657242	**Variance**	32276.7245
**Skewness**	1.35136353	**Kurtosis**	2.22099672
**Uncorrected SS**	6955505.35	**Corrected SS**	710087.94
**Coeff Variation**	34.4768763	**Std Error Mean**	37.4611245

Quantiles (Definition 5)	
**Quantile**	**Estimate**
**100% Max**	1029.50
**99%**	1029.50
**95%**	898.25
**90%**	755.00
**75% Q3**	561.00
**50% Median**	485.00
**25% Q1**	406.00
**10%**	357.00
**5%**	312.60
**1%**	260.00
**0% Min**	260.00

The NPAR1WAY Procedure
Treat=cd_4

Wilcoxon Scores (Rank Sums) for Variable HCV Classified by Variable time_0_vs_24

time_0_vs_24	N	Sum of Scores	Expected Under H0	Std Dev Under H0	Mean Score
**0**	23	389.0	540.50	45.518311	16.913043
**24**	23	692.0	540.50	45.518311	30.086957

Wilcoxon Two-Sample Test

**Statistic**	389.0000
**Normal Approximation**	
**Z**	-3.3173
**One-Sided Pr < Z**	0.0005
**Two-Sided Pr > |Z|**	0.0009
**t Approximation**	
**One-Sided Pr < Z**	0.0009
**Two-Sided Pr > |Z|**	0.0018

Z includes a continuity correction of 0.5.

Kruskal-Wallis Test

**Chi-Square**	11.0778
**DF**	1
**Pr > Chi-Square**	0.0009

The NPAR1WAY Procedure
Treat=cd_4

Wilcoxon Scores (Rank Sums) for Variable HCV Classified by Variable time_12_vs_24

time_12_vs_24	N	Sum of Scores	Expected Under H0	Std Dev Under H0	Mean Score
**12**	23	430.0	540.50	45.518311	18.695652
**24**	23	651.0	540.50	45.518311	28.304348

Wilcoxon Two-Sample Test

**Statistic**	430.0000
**Normal Approximation**	
**Z**	-2.4166
**One-Sided Pr < Z**	0.0078
**Two-Sided Pr > |Z|**	0.0157
**t Approximation**	
**One-Sided Pr < Z**	0.0099
**Two-Sided Pr > |Z|**	0.0198

Z includes a continuity correction of 0.5.

Kruskal-Wallis Test

**Chi-Square**	5.8932
**DF**	1
**Pr > Chi-Square**	0.0152

The UNIVARIATE Procedure
Variable: HCV (HCV)
cd_4:cd_8_naïve

Moments			
**N**	21	**Sum Weights**	21
**Mean**	0.37952381	**Sum Observations**	7.97
**Std Deviation**	0.30292699	**Variance**	0.09176476
**Skewness**	1.4991632	**Kurtosis**	1.90482872
**Uncorrected SS**	4.8601	**Corrected SS**	1.83529524
**Coeff Variation**	79.8176513	**Std Error Mean**	0.06610409

Quantiles (Definition 5)	
**Quantile**	**Estimate**
**100% Max**	1.20
**99%**	1.20
**95%**	0.93
**90%**	0.93
**75% Q3**	0.47
**50% Median**	0.32
**25% Q1**	0.16
**10%**	0.13
**5%**	0.06
**1%**	0.04
**0% Min**	0.04

The SAS System

The UNIVARIATE Procedure
Variable: HCV (HCV)
Treat=cd_4:cd_8_time_12_months

Moments			
**N**	23	**Sum Weights**	23
**Mean**	0.46947705	**Sum Observations**	10.797972
**Std Deviation**	0.25162292	**Variance**	0.06331409
**Skewness**	1.14999088	**Kurtosis**	2.20756216
**Uncorrected SS**	6.46231007	**Corrected SS**	1.39291006
**Coeff Variation**	53.5964265	**Std Error Mean**	0.05246701

Quantiles (Definition 5)	
**Quantile**	**Estimate**
**100% Max**	1.202538
**99%**	1.202538
**95%**	0.929559
**90%**	0.697546
**75% Q3**	0.561721
**50% Median**	0.481043
**25% Q1**	0.288509
**10%**	0.181119
**5%**	0.167385
**1%**	0.105423
**0% Min**	0.105423

The NPAR1WAY Procedure
Treat=cd_4_8

Wilcoxon Scores (Rank Sums) for Variable HCV Classified by Variable time_0_vs_12

time_0_vs_12	N	Sum of Scores	Expected Under H0	Std Dev Under H0	Mean Score
0	21	395.0	472.50	42.555784	18.809524
12	23	595.0	517.50	42.555784	25.869565

Average scores were used for ties.

Wilcoxon Two-Sample Test

Statistic	395.0000
**Normal Approximation**	
Z	-1.8094
One-Sided Pr < Z	0.0352
Two-Sided Pr > |Z|	0.0704
**t Approximation**	
One-Sided Pr < Z	0.0387
Two-Sided Pr > |Z|	0.0774

Z includes a continuity correction of 0.5.

Kruskal-Wallis Test

Chi-Square	3.3165
DF	1
Pr > Chi-Square	0.0686

The SAS System

The UNIVARIATE Procedure
Variable: HCV (HCV)
Treat=cd_4:cd_8_time_24_months

Moments			
**N**	23	**Sum Weights**	23
**Mean**	0.5903869	**Sum Observations**	13.5788988
**Std Deviation**	0.24704325	**Variance**	0.06103037
**Skewness**	1.09898667	**Kurtosis**	2.00357549
**Uncorrected SS**	9.3594721	**Corrected SS**	1.34266812
**Coeff Variation**	41.8442977	**Std Error Mean**	0.05151208

Quantiles (Definition 5)	
**Quantile**	**Estimate**
**100% Max**	1.313138
**99%**	1.313138
**95%**	0.935780
**90%**	0.872832
**75% Q3**	0.708029
**50% Median**	0.496272
**25% Q1**	0.433754
**10%**	0.332751
**5%**	0.317933
**1%**	0.185464
**0% Min**	0.185464

The NPAR1WAY Procedure
Treat=cd_4_8

Wilcoxon Scores (Rank Sums) for Variable HCV Classified by Variable time_0_vs_24

time_0_vs_24	N	Sum of Scores	Expected Under H0	Std Dev Under H0	Mean Score
**0**	21	348.0	472.50	42.555784	16.571429
**24**	23	642.0	517.50	42.555784	27.913043

Average scores were used for ties.

Wilcoxon Two-Sample Test

**Statistic**	348.0000
**Normal Approximation**	
**Z**	-2.9138
**One-Sided Pr < Z**	0.0018
**Two-Sided Pr > |Z|**	0.0036
**t Approximation**	
**One-Sided Pr < Z**	0.0028
**Two-Sided Pr > |Z|**	0.0056

Z includes a continuity correction of 0.5.

Kruskal-Wallis Test

**Chi-Square**	8.5590
**DF**	1
**Pr > Chi-Square**	0.0034

The NPAR1WAY Procedure
Treat=cd_4_8

Wilcoxon Scores (Rank Sums) for Variable HCV Classified by Variable time_12_vs_24

time_12_vs_24	N	Sum of Scores	Expected Under H0	Std Dev Under H0	Mean Score
**12**	23	463.0	540.50	45.518311	20.130435
**24**	23	618.0	540.50	45.518311	26.869565

Wilcoxon Two-Sample Test

**Statistic**	463.0000
**Normal Approximation**	
**Z**	-1.6916
**One-Sided Pr < Z**	0.0454
**Two-Sided Pr > |Z|**	0.0907
**t Approximation**	
**One-Sided Pr < Z**	0.0488
**Two-Sided Pr > |Z|**	0.0976

Z includes a continuity correction of 0.5.

Kruskal-Wallis Test

**Chi-Square**	2.8989
**DF**	1
**Pr > Chi-Square**	0.0886

The UNIVARIATE Procedure
Variable: HCV (HCV)
cv_log_naïve

Moments			
**N**	19	**Sum Weights**	19
**Mean**	3.84786664	**Sum Observations**	73.1094661
**Std Deviation**	1.63562441	**Variance**	2.6752672
**Skewness**	-0.1319716	**Kurtosis**	-1.4781717
**Uncorrected SS**	329.470285	**Corrected SS**	48.1548095
**Coeff Variation**	42.5073049	**Std Error Mean**	0.37523797

Quantiles (Definition 5)	
**Quantile**	**Estimate**
**100% Max**	6.00000
**99%**	6.00000
**95%**	6.00000
**90%**	6.00000
**75% Q3**	5.35218
**50% Median**	4.05258
**25% Q1**	1.69897
**10%**	1.69897
**5%**	1.69897
**1%**	1.69897
**0% Min**	1.69897

The SAS System

The UNIVARIATE Procedure
Variable: HCV (HCV)
Treat=cv_log time_12_months

Moments			
**N**	23	**Sum Weights**	23
**Mean**	1.8309835	**Sum Observations**	42.1126206
**Std Deviation**	0.51657329	**Variance**	0.26684797
**Skewness**	2.457642	**Kurtosis**	6.03110656
**Uncorrected SS**	82.9781689	**Corrected SS**	5.87065527
**Coeff Variation**	28.2128862	**Std Error Mean**	0.10771298

Quantiles (Definition 5)	
**Quantile**	**Estimate**
**100% Max**	3.58422
**99%**	3.58422
**95%**	2.83948
**90%**	2.73640
**75% Q3**	1.69897
**50% Median**	1.69897
**25% Q1**	1.69897
**10%**	1.39794
**5%**	1.39794
**1%**	1.30103
**0% Min**	1.30103

The NPAR1WAY Procedure
Treat=cv_log

Wilcoxon Scores (Rank Sums) for Variable HCV Classified by Variable time_0_vs_12

time_0_vs_12	N	Sum of Scores	Expected Under H0	Std Dev Under H0	Mean Score
0	19	567.0	408.50	37.016974	29.842105
12	23	336.0	494.50	37.016974	14.608696

Average scores were used for ties.

Wilcoxon Two-Sample Test

**Statistic**	567.0000
**Normal Approximation**	
**Z**	4.2683
**One-Sided Pr > Z**	<.0001
**Two-Sided Pr > |Z|**	<.0001
**t Approximation**	
**One-Sided Pr > Z**	<.0001
**Two-Sided Pr > |Z|**	0.0001

Z includes a continuity correction of 0.5.

Kruskal-Wallis Test

**Chi-Square**	18.3340
**DF**	1
**Pr > Chi-Square**	<.0001

The SAS System

The UNIVARIATE Procedure
Variable: HCV (HCV)
Treat=cv_log time_24_months

Moments			
**N**	23	**Sum Weights**	23
**Mean**	1.63978574	**Sum Observations**	37.7150719
**Std Deviation**	0.32821062	**Variance**	0.10772221
**Skewness**	2.58708864	**Kurtosis**	10.3701658
**Uncorrected SS**	64.2145257	**Corrected SS**	2.36988866
**Coeff Variation**	20.0154577	**Std Error Mean**	0.06843665

Quantiles (Definition 5)	
**Quantile**	**Estimate**
**100% Max**	2.91116
**99%**	2.91116
**95%**	1.77085
**90%**	1.69897
**75% Q3**	1.69897
**50% Median**	1.69897
**25% Q1**	1.39794
**10%**	1.30103
**5%**	1.30103
**1%**	1.30103
**0% Min**	1.30103

The NPAR1WAY Procedure
Treat=cv_log

Wilcoxon Scores (Rank Sums) for Variable HCV Classified by Variable time_0_vs_24

time_0_vs_24	N	Sum of Scores	Expected Under H0	Std Dev Under H0	Mean Score
**0**	19	583.50	408.50	37.949589	30.710526
**24**	23	319.50	494.50	37.949589	13.891304

Average scores were used for ties.

Wilcoxon Two-Sample Test

**Statistic**	583.5000
**Normal Approximation**	
**Z**	4.5982
**One-Sided Pr > Z**	<.0001
**Two-Sided Pr > |Z|**	<.0001
**t Approximation**	
**One-Sided Pr > Z**	<.0001
**Two-Sided Pr > |Z|**	<.0001

Z includes a continuity correction of 0.5.

Kruskal-Wallis Test

**Chi-Square**	21.2648
**DF**	1
**Pr > Chi-Square**	<.0001

The NPAR1WAY Procedure
Treat=cv_log

Wilcoxon Scores (Rank Sums) for Variable HCV Classified by Variable time_12_vs_24

time_12_vs_24	N	Sum of Scores	Expected Under H0	Std Dev Under H0	Mean Score
**12**	23	597.50	540.50	39.336864	25.978261
**24**	23	483.50	540.50	39.336864	21.021739

Average scores were used for ties.

Wilcoxon Two-Sample Test

**Statistic**	597.5000
**Normal Approximation**	
**Z**	1.4363
**One-Sided Pr > Z**	0.0755
**Two-Sided Pr > |Z|**	0.1509
**t Approximation**	
**One-Sided Pr > Z**	0.0789
**Two-Sided Pr > |Z|**	0.1578

Z includes a continuity correction of 0.5.

Kruskal-Wallis Test

**Chi-Square**	2.0997
**DF**	1
**Pr > Chi-Square**	0.1473

The SAS System

The UNIVARIATE Procedure
Variable: hepatitis (hepatitis)
cd_4_naïve

Moments			
**N**	47	**Sum Weights**	47
**Mean**	353.893617	**Sum Observations**	16633
**Std Deviation**	235.479182	**Variance**	55450.445
**Skewness**	1.50883137	**Kurtosis**	2.83466909
**Uncorrected SS**	8437033	**Corrected SS**	2550720.47
**Coeff Variation**	66.5395391	**Std Error Mean**	34.3481688

Quantiles (Definition 5)	
**Quantile**	**Estimate**
**100% Max**	1171
**99%**	1171
**95%**	869
**90%**	697
**75% Q3**	467
**50% Median**	312
**25% Q1**	202
**10%**	128
**5%**	67
**1%**	30
**0% Min**	30

The UNIVARIATE Procedure
Variable: hepatitis (hepatitis)
Treat=cd_4_time_12_months

Moments			
**N**	47	**Sum Weights**	47
**Mean**	462.387289	**Sum Observations**	21732.2026
**Std Deviation**	224.311503	**Variance**	50315.6504
**Skewness**	1.20229569	**Kurtosis**	1.52505047
**Uncorrected SS**	12363214.1	**Corrected SS**	2314519.92
**Coeff Variation**	48.5116067	**Std Error Mean**	32.7191955

Quantiles (Definition 5)

Quantile	Estimate
**100% Max**	1103.000
**99%**	1103.000
**95%**	1009.330
**90%**	706.000
**75% Q3**	568.500
**50% Median**	435.333
**25% Q1**	266.330
**10%**	233.000
**5%**	208.000
**1%**	191.000
**0% Min**	191.000

The NPAR1WAY Procedure
Treat=cd_4

Wilcoxon Scores (Rank Sums) for Variable hepatitis Classified by Variable time_0_vs_12

time_0_vs_12	N	Sum of Scores	Expected Under H0	Std Dev Under H0	Mean Score
0	47	1877.0	2232.50	132.240932	39.936170
12	47	2588.0	2232.50	132.240932	55.063830

Average scores were used for ties.

Wilcoxon Two-Sample Test

Statistic	1877.0000
**Normal Approximation**	
Z	-2.6845
One-Sided Pr < Z	0.0036
Two-Sided Pr > |Z|	0.0073
**t Approximation**	
One-Sided Pr < Z	0.0043
Two-Sided Pr > |Z|	0.0086

Z includes a continuity correction of 0.5.

Kruskal-Wallis Test

Chi-Square	7.2268
DF	1
Pr > Chi-Square	0.0072

The SAS System

The UNIVARIATE Procedure
Variable: hepatitis (hepatitis)
Treat=cd_4_time_24_months

Moments			
**N**	47	**Sum Weights**	47
**Mean**	560.042394	**Sum Observations**	26321.9925
**Std Deviation**	207.748795	**Variance**	43159.562
**Skewness**	0.61617295	**Kurtosis**	0.24658514
**Uncorrected SS**	16726771.6	**Corrected SS**	1985339.85
**Coeff Variation**	37.0951909	**Std Error Mean**	30.3032763

Quantiles (Definition 5)	
**Quantile**	**Estimate**
**100% Max**	1127.00
**99%**	1127.00
**95%**	908.00
**90%**	834.66
**75% Q3**	662.00
**50% Median**	554.00
**25% Q1**	406.00
**10%**	312.60
**5%**	279.50
**1%**	170.00
**0% Min**	170.00

The SAS System

The NPAR1WAY Procedure
Treat=cd_4

Wilcoxon Scores (Rank Sums) for Variable hepatitis Classified by Variable time_0_vs_24

time_0_vs_24	N	Sum of Scores	Expected Under H0	Std Dev Under H0	Mean Score
**0**	47	1596.0	2232.50	132.241409	33.957447
**24**	47	2869.0	2232.50	132.241409	61.042553

Average scores were used for ties.

Wilcoxon Two-Sample Test

**Statistic**	1596.0000
**Normal Approximation**	
**Z**	-4.8094
**One-Sided Pr < Z**	<.0001
**Two-Sided Pr > |Z|**	<.0001
**t Approximation**	
**One-Sided Pr < Z**	<.0001
**Two-Sided Pr > |Z|**	<.0001

Z includes a continuity correction of 0.5.

Kruskal-Wallis Test

**Chi-Square**	23.1666
**DF**	1
**Pr > Chi-Square**	<.0001

The NPAR1WAY Procedure
Treat=cd_4

Wilcoxon Scores (Rank Sums) for Variable hepatitis Classified by Variable time_12_vs_24

time_12_vs_24	N	Sum of Scores	Expected Under H0	Std Dev Under H0	Mean Score
**12**	47	1903.50	2232.50	132.240932	40.50
**24**	47	2561.50	2232.50	132.240932	54.50

Average scores were used for ties.

Wilcoxon Two-Sample Test

**Statistic**	1903.5000
**Normal Approximation**	
**Z**	-2.4841
**One-Sided Pr < Z**	0.0065
**Two-Sided Pr > |Z|**	0.0130
**t Approximation**	
**One-Sided Pr < Z**	0.0074
**Two-Sided Pr > |Z|**	0.0148

Z includes a continuity correction of 0.5.

Kruskal-Wallis Test

**Chi-Square**	6.1896
**DF**	1
**Pr > Chi-Square**	0.0129

The SAS System

The UNIVARIATE Procedure
Variable: hepatitis (hepatitis)
cd_4:cd_8_naïve

Moments			
**N**	47	**Sum Weights**	47
**Mean**	0.39957447	**Sum Observations**	18.78
**Std Deviation**	0.3236474	**Variance**	0.10474764
**Skewness**	1.69613778	**Kurtosis**	3.2478416
**Uncorrected SS**	12.3224	**Corrected SS**	4.81839149
**Coeff Variation**	80.9980187	**Std Error Mean**	0.04720883

Quantiles (Definition 5)	
**Quantile**	**Estimate**
**100% Max**	1.59
**99%**	1.59
**95%**	0.96
**90%**	0.93
**75% Q3**	0.50
**50% Median**	0.30
**25% Q1**	0.17
**10%**	0.12
**5%**	0.06
**1%**	0.04
**0% Min**	0.04

The SAS System

The UNIVARIATE Procedure
Variable: hepatitis (hepatitis)
Treat=cd_4:cd_8_time_12_months

Moments			
**N**	47	**Sum Weights**	47
**Mean**	0.52791123	**Sum Observations**	24.8118277
**Std Deviation**	0.33865149	**Variance**	0.11468483
**Skewness**	1.7241357	**Kurtosis**	3.90472742
**Uncorrected SS**	18.3739449	**Corrected SS**	5.2755024
**Coeff Variation**	64.1493259	**Std Error Mean**	0.0493974

Quantiles (Definition 5)	
**Quantile**	**Estimate**
**100% Max**	1.807882
**99%**	1.807882
**95%**	1.202538
**90%**	0.992703
**75% Q3**	0.632539
**50% Median**	0.481043
**25% Q1**	0.291814
**10%**	0.188291
**5%**	0.167385
**1%**	0.105423
**0% Min**	0.105423

The NPAR1WAY Procedure
Treat=cd_4_8

Wilcoxon Scores (Rank Sums) for Variable hepatitis Classified by Variable time_0_vs_12

time_0_vs_12	N	Sum of Scores	Expected Under H0	Std Dev Under H0	Mean Score
**0**	47	1901.0	2232.50	132.236154	40.446809
**12**	47	2564.0	2232.50	132.236154	54.553191

Average scores were used for ties.

Wilcoxon Two-Sample Test

**Statistic**	1901.0000
**Normal Approximation**	
**Z**	-2.5031
**One-Sided Pr < Z**	0.0062
**Two-Sided Pr > |Z|**	0.0123
**t Approximation**	
**One-Sided Pr < Z**	0.0070
**Two-Sided Pr > |Z|**	0.0141

Z includes a continuity correction of 0.5.

Kruskal-Wallis Test

**Chi-Square**	6.2844
**DF**	1
**Pr > Chi-Square**	0.0122

The SAS System

The UNIVARIATE Procedure
Variable: hepatitis (hepatitis)
Treat=cd_4:cd_8_time_24_months

Moments			
**N**	47	**Sum Weights**	47
**Mean**	0.65347821	**Sum Observations**	30.713476
**Std Deviation**	0.31192005	**Variance**	0.09729412
**Skewness**	1.20759219	**Kurtosis**	2.36382749
**Uncorrected SS**	24.5461169	**Corrected SS**	4.4755295
**Coeff Variation**	47.7322804	**Std Error Mean**	0.04549822

Quantiles (Definition 5)

Quantile	Estimate
**100% Max**	1.739198
**99%**	1.739198
**95%**	1.313138
**90%**	0.964548
**75% Q3**	0.855786
**50% Median**	0.614629
**25% Q1**	0.458201
**10%**	0.331034
**5%**	0.232814
**1%**	0.173293
**0% Min**	0.173293

The SAS System

The NPAR1WAY Procedure
Treat=cd_4_8

Wilcoxon Scores (Rank Sums) for Variable hepatitis Classified by Variable time_0_24

time_0_24	N	Sum of Scores	Expected Under H0	Std Dev Under H0	Mean Score
**0**	47	1650.0	2232.50	132.236154	35.106383
**24**	47	2815.0	2232.50	132.236154	59.893617

Average scores were used for ties.

Wilcoxon Two-Sample Test

**Statistic**	1650.0000
**Normal Approximation**	
**Z**	-4.4012
**One-Sided Pr < Z**	<.0001
**Two-Sided Pr > |Z|**	<.0001
**t Approximation**	
**One-Sided Pr < Z**	<.0001
**Two-Sided Pr > |Z|**	<.0001

Z includes a continuity correction of 0.5.

Kruskal-Wallis Test

**Chi-Square**	19.4040
**DF**	1
**Pr > Chi-Square**	<.0001

The SAS System

The NPAR1WAY Procedure
Treat=cd_4_8

Wilcoxon Scores (Rank Sums) for Variable hepatitis Classified by Variable time_12_vs_24

time_12_vs_24	N	Sum of Scores	Expected Under H0	Std Dev Under H0	Mean Score
12	47	1912.0	2232.50	132.241887	40.680851
24	47	2553.0	2232.50	132.241887	54.319149

Wilcoxon Two-Sample Test

Statistic	1912.0000
Normal Approximation	
Z	-2.4198
One-Sided Pr < Z	0.0078
Two-Sided Pr > |Z|	0.0155
t Approximation	
One-Sided Pr < Z	0.0087
Two-Sided Pr > |Z|	0.0175

Z includes a continuity correction of 0.5.

Kruskal-Wallis Test

Chi-Square	5.8738
DF	1
Pr > Chi-Square	0.0154

The UNIVARIATE Procedure
Variable: hepatitis (hepatitis)
cv_log_naïve

Moments			
**N**	39	**Sum Weights**	39
**Mean**	3.20837	**Sum Observations**	77.0008801
**Std Deviation**	1.1555229	**Variance**	1.33523317
**Skewness**	-0.2632938	**Kurtosis**	-1.6299519
**Uncorrected SS**	277.757677	**Corrected SS**	30.7103629
**Coeff Variation**	36.0158865	**Std Error Mean**	0.23587012

Quantiles (Definition 5)	
**Quantile**	**Estimate**
**100% Max**	4.69512
**99%**	4.69512
**95%**	4.60977
**90%**	4.53667
**75% Q3**	4.21188
**50% Median**	3.55601
**25% Q1**	1.69897
**10%**	1.69897
**5%**	1.69897
**1%**	1.69897
**0% Min**	1.69897

The SAS System

The UNIVARIATE Procedure
Variable: hepatitis (hepatitis)
Treat=cv_log time_12_months

Moments			
**N**	47	**Sum Weights**	47
**Mean**	1.83608391	**Sum Observations**	86.2959436
**Std Deviation**	0.43927616	**Variance**	0.19296354
**Skewness**	2.18154879	**Kurtosis**	5.34636267
**Uncorrected SS**	167.322916	**Corrected SS**	8.87632305
**Coeff Variation**	23.9246234	**Std Error Mean**	0.06407501

Quantiles (Definition 5)	
**Quantile**	**Estimate**
**100% Max**	3.58422
**99%**	3.58422
**95%**	2.80889
**90%**	2.60206
**75% Q3**	1.77815
**50% Median**	1.69897
**25% Q1**	1.69897
**10%**	1.39794
**5%**	1.30103
**1%**	1.30103
**0% Min**	1.30103

The NPAR1WAY Procedure
Treat=cv_log

Wilcoxon Scores (Rank Sums) for Variable hepatitis Classified by Variable time_0_vs_12

time_0_vs_12	N	Sum of Scores	Expected Under H0	Std Dev Under H0	Mean Score
**0**	39	1233.0	864.0	78.027963	51.375000
**12**	47	1323.0	1692.0	78.027963	28.148936

Average scores were used for ties.

Wilcoxon Two-Sample Test

**Statistic**	1233.0000
**Normal Approximation**	
**Z**	4.7227
**One-Sided Pr > Z**	<.0001
**Two-Sided Pr > |Z|**	<.0001
**t Approximation**	
**One-Sided Pr > Z**	<.0001
**Two-Sided Pr > |Z|**	<.0001

Z includes a continuity correction of 0.5.

Kruskal-Wallis Test

**Chi-Square**	22.3641
**DF**	1
**Pr > Chi-Square**	<.0001

The UNIVARIATE Procedure
Variable: hepatitis (hepatitis)
Treat=cv_log time_24_months

Moments			
**N**	47	**Sum Weights**	47
**Mean**	1.63999142	**Sum Observations**	77.0795966
**Std Deviation**	0.33100244	**Variance**	0.10956262
**Skewness**	2.55031731	**Kurtosis**	9.3591623
**Uncorrected SS**	131.449757	**Corrected SS**	5.03988036
**Coeff Variation**	20.1831814	**Std Error Mean**	0.04828167

Quantiles (Definition 5)	
**Quantile**	**Estimate**
**100% Max**	2.99056
**99%**	2.99056
**95%**	1.83251
**90%**	1.75587
**75% Q3**	1.69897
**50% Median**	1.69897
**25% Q1**	1.39794
**10%**	1.30103
**5%**	1.30103
**1%**	1.30103
**0% Min**	1.30103

The NPAR1WAY Procedure
Treat=cv_log

Wilcoxon Scores (Rank Sums) for Variable hepatitis Classified by Variable time_0_vs_24

time_0_vs_24	N	Sum of Scores	Expected Under H0	Std Dev Under H0	Mean Score
**0**	39	1296.0	864.0	77.896960	54.000000
**24**	47	1260.0	1692.0	77.896960	26.808511

Average scores were used for ties.

Wilcoxon Two-Sample Test

**Statistic**	1296.0000
**Normal Approximation**	
**Z**	5.5394
**One-Sided Pr > Z**	<.0001
**Two-Sided Pr > |Z|**	<.0001
**t Approximation**	
**One-Sided Pr > Z**	<.0001
**Two-Sided Pr > |Z|**	<.0001

Z includes a continuity correction of 0.5.

Kruskal-Wallis Test

**Chi-Square**	30.7558
**DF**	1
**Pr > Chi-Square**	<.0001

The NPAR1WAY Procedure
Treat=cv_log

Wilcoxon Scores (Rank Sums) for Variable hepatitis Classified by Variable time_12_vs_24

time_12_vs_24	N	Sum of Scores	Expected Under H0	Std Dev Under H0	Mean Score
**12**	47	2552.0	2232.50	120.312575	54.297872
**24**	47	1913.0	2232.50	120.312575	40.702128

Average scores were used for ties.

Wilcoxon Two-Sample Test

**Statistic**	2552.0000
**Normal Approximation**	
**Z**	2.6514
**One-Sided Pr > Z**	0.0040
**Two-Sided Pr > |Z|**	0.0080
**t Approximation**	
**One-Sided Pr > Z**	0.0047
**Two-Sided Pr > |Z|**	0.0094

Z includes a continuity correction of 0.5.

Kruskal-Wallis Test

**Chi-Square**	7.0521
**DF**	1
**Pr > Chi-Square**	0.0079

Frequency
Col Pct

Table of cd4_diagnosis_transformed by naïve	<200	200-500	>500	Total
HIV-HBV	5<br>20.84	15<br>62.50	4<br>16.66	24
HIV-HCV	6<br>26.09	13<br>56.52	4<br>17.39	23
HIV-hepatitis	11<br>23.41	28<br>59.57	8<br>17.02	47
Total	22	56	16	94

Frequency
Col Pct

Table of cd4_diagnosis_transformed by 12 months	<200	200-500	>500	Total
HIV-HBV	1<br>4.17	11<br>45.83	12<br>50.00	24
HIV-HCV	1<br>4.17	16<br>69.57	6<br>26.08	23
HIV-hepatitis	2<br>4.26	27<br>57.45	18<br>38.29	47
Total	4	54	36	94

Frequency
Col Pct

Table of cd4_diagnosis_transformed by 24 months	<200	200-500	>500	Total
HIV-HBV	1<br>4.17	7<br>29.17	16<br>66.66	24
HIV-HCV	0<br>0.00	12<br>52.17	11<br>47.83	23
HIV-hepatitis	1<br>2.13	19<br>40.43	27<br>57.44	47
Total	4	54	36	94

Statistics for Table of cd4_diagnosis
HIV-HBV time naïve vs 12

Statistic	DF	Value	Prob
Chi-Square	2	7.282	0.0262

Statistics for Table of cd4_diagnosis

HIV-HBV time naïve vs 24

Statistic	DF	Value	Prob
Chi-Square	2	12.776	0.0017

Statistics for Table of cd4_diagnosis
HIV-HBV time 12 vs 24

Statistic	DF	Value	Prob
Chi-Square	2	1.460	0.4818

Statistics for Table of cd4_diagnosis
HIV-HCV time naïve vs 12

Statistic	DF	Value	Prob
Chi-Square	2	4.282	0.1176

Statistics for Table of cd4_diagnosis
HIV-HCV time naïve vs 24

Statistic	DF	Value	Prob
Chi-Square	2	9.307	0.0095

Statistics for Table of cd4_diagnosis
HIV-HCV time 12 vs 24

Statistic	DF	Value	Prob
Chi-Square	2	3.042	0.2185

Statistics for Table of cd4_diagnosis
hepatitis time naïve vs 12

Statistic	DF	Value	Prob
Chi-Square	2	10.095	0.0064

Statistics for Table of cd4_diagnosis
hepatitis time naïve vs 24

Statistic	DF	Value	Prob
Chi-Square	2	20.371	<.0001

Statistics for Table of cd4_diagnosis
hepatitis time 12 vs 24

Statistic	DF	Value	Prob
Chi-Square	2	3.525	0.1716
